# Heck- and Suzuki-coupling approaches to novel hydroquinone inhibitors of calcium ATPase

**DOI:** 10.3762/bjoc.15.94

**Published:** 2019-04-24

**Authors:** Robert J Kempton, Taylor A Kidd-Kautz, Soizic Laurenceau, Stefan Paula

**Affiliations:** 1 Department of Chemistry and Biochemistry, Northern Kentucky University, Nunn Drive, Highland Heights, KY 41099, USA; 2 Department of Chemistry, Purdue University, Oval Drive, West Lafayette, IN 47907, USA

**Keywords:** calcium ATPase inhibitors, Heck- and Suzuki-coupling reactions, hydroquinones, prostate cancer, tethered amino acid

## Abstract

In this study, we explored Heck- and Suzuki-coupling methodology to modify the template 2,5-di-*tert*-butylhydroquinone (BHQ, **2**), an inhibitor of the enzyme sarco/endoplasmic reticulum calcium ATPase (SERCA). We found that by utilizing Suzuki coupling, we could successfully attach a six-carbon tether to BHQ that terminated in a leucine moiety to obtain target **14**. Similar to related compounds based on the structure of the natural product thapsigargin, **14** displayed inhibitory potency against SERCA activity. This makes **14** a suitable candidate for the future attachment of a deactivating peptide to convey specificity for prostate cancer cells.

## Introduction

**Figure 1:**
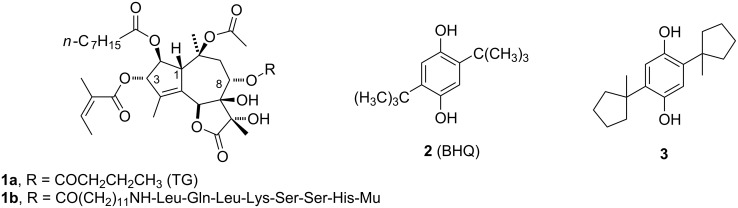
Thapsigargin- and hydroquinone-based SERCA inhibitors.

Sarco/endoplasmic reticulum calcium ATPase (SERCA) is an integral protein that resides in the membrane of the sarcoplasmic reticulum (SR) within muscle cells. It transfers Ca^2+^ from the cytosol to the lumen of the SR at the expense of ATP hydrolysis. Specific inhibitors of SERCA are of significance to human health because of their well-documented value as research tools and their potential as novel anticancer agents [1–2]. The natural product thapsigargin (TG, **1a**, Figure 1) is one of the most frequently used SERCA inhibitors because of its high specificity and potency. Both cancerous and healthy cells undergo apoptosis after exposure to low concentrations of TG, making TG a highly potent but nonselective cytotoxic agent. The problem of concomitant toxicity to healthy cells has been circumvented by attaching a short peptide (His-Ser-Ser-Lys-Leu-Gln-Leu) to a tether at TG’s C-8 position (**1b**). This modification renders the inhibitor inactive [3]. Prostate cancer cells produce on their surface the serine protease PSA (prostate-specific antigen) that is capable of cleaving the peptide bond between Gln and Leu, thereby producing an active TG analogue that can enter the cancer cell and kill it by triggering apoptosis. Apoptosis occurs as the result of elevated cytosolic calcium levels, which are caused by inhibiting SERCA and preventing it from loading intracellular calcium stores. No other major proteases share the specificity of PSA, which prevents premature inhibitor activation in healthy cells. Moreover, as PSA is deactivated by inhibitors present in the blood serum, potential detrimental effects on other tissues are avoided. Compound **1b** was found to be selectively toxic to PSA-producing prostate cancer cells as well as in animal studies at submicromolar concentrations [3].

Unfortunately, TG has been difficult to obtain: extraction yields from natural sources are low and the total synthesis of TG required 42 synthetic steps [4]. More recently, two shorter, scalable total syntheses of TG have been reported [5–6].

As an alternative to TG, the structurally much less complex inhibitor 2,5-di-*tert*-butylhydroquinone (BHQ, **2**, Figure 1) has a somewhat lower inhibitory potency than TG (400 nM versus low nanomolar range), but offers advantages such as ease of synthesis and greatly reduced cost. Recent synthetic work from our group has shown that the *tert*-butyl groups of BHQ can be replaced by a variety of alkyl and cycloalkyl groups with minimal reduction in potency [7–9]. In fact, the 2,5-bis(1-methylcyclopentyl) analogue of BHQ **3** has an IC_50_ value of 500 nM, which is comparable to BHQ’s value of 400 nM. Having available tethered SERCA inhibitors that are not based on the structure of TG could be beneficial for future in vivo trials because of the greater flexibility afforded by the structurally less complex BHQ template. Furthermore, using a BHQ template could be a valuable alternative to TG-based compounds, should the latter encounter problems such as low bioavailability or drug resistance.

The first step towards the development of BHQ-based compounds of therapeutic value is the establishment of a synthetic route to BHQ analogues with a tether that could serve as the basis for future efforts aimed at the attachment of deactivating peptides. The rationale is analogous to the Denmeade group’s strategy for circumventing TG’s toxicity to healthy cells by selective transesterification of the ester group at C-8 of TG, replacing the natural butanoyl group at that position with an ester moiety terminating in a primary amine.

Here, we describe our efforts to use transition metal cross-coupling reactions (Suzuki [10] and Heck [11]) to modify BHQ with a side chain terminating with a free primary amine that could serve as an attachment point for a peptide group (see **1b** above). We had anticipated that the availability and versatility of cross-coupling reactions would allow for the introduction of side chains of varying lengths and functionalities [10]. In this paper, we report on the synthesis of **14**, a BHQ analogue and active SERCA inhibitor containing a side chain terminating in a leucine moiety.

## Results and Discussion

**Scheme 1:**
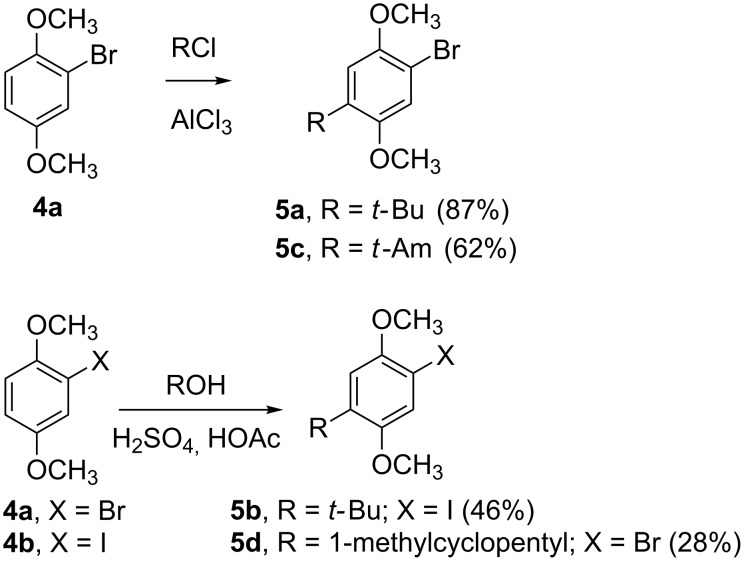
Friedel–Crafts alkylation of **4**.

The starting materials for the syntheses were the halides **4a**,**b** (Scheme 1). Bromide **4a** is commercially available. We investigated several ways to prepare the corresponding iodide **4b** from 1,4-dimethoxybenzene, among them iodine in the presence of trichlorocyanuric acid and silica gel [9], I_2_/periodic acid [10], I_2_/silfen [11], *N*-iodosuccinimide [12], and potassium iodide/potassium iodate [13]. In our hands the best yields of **4b** were obtained using iodine in the presence of silfen [11]. Alkylation of **4** under typical Friedel–Crafts conditions gave **5**. In the case of **5a**, the ^1^H NMR of the crude product, while clearly showing that it was the desired 4-substituted isomer (^1^H singlets at δ 6.89 and 7.02), also exhibited an impurity (doublets at δ 6.83 and 7.12, *J* = 3.0 Hz) suggesting the presence of a small amount of the 3-*tert*-butyl isomer. The corresponding iodo analogue **5b** was prepared following the procedure of Hayashi [14]. Although both halides **5a** and **5b** were now available to us, subsequent experiments determined that the iodo analogue offered us no advantage over the more accessible bromo analogue **5a** in cross-coupling reactions.

**Scheme 2:**
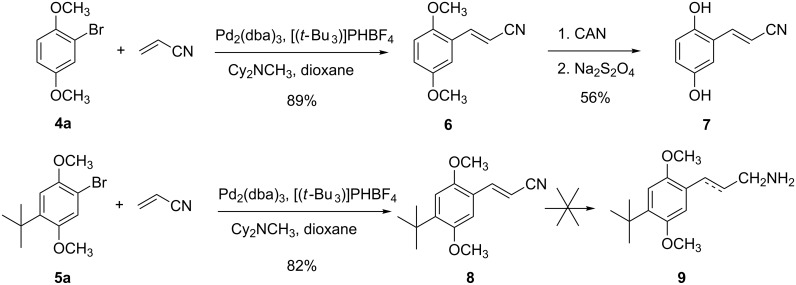
Heck cross-coupling reactions.

Heck cross coupling [15] of **4a** and **5a** with acrylonitrile in the presence of Pd_2_(dba)_3_, [(*t*-Bu)_3_PH]BF_4_ and Cy_2_NCH_3_ in dioxane, following the procedure of Fu [16–17], gave the coupled products **6** and **8**, respectively, in yields of 89% and 82%, respectively (Scheme 2). In both cases, the products were a mixture of *E-* and *Z-*isomers, with an *E*/*Z* ratio of ca. 85:15 based on ^1^H NMR. The pure *E*-isomers could be obtained by recrystallization from hexane. ^1^H NMR spectra of the recrystallized products exhibited doublets at δ 6.03 and 7.61 (*J* = 16.9 Hz) and 6.01 and 7.57 (*J* = 17.0 Hz) for **6** and **8**, respectively, confirming the *E*-stereochemistry. Attempts to convert the two methoxy groups of **6** and **8** to hydroxy groups (e.g., **7**) in a single step using BBr_3_·S(CH_3_)_2_ [18] or (CH_3_)_3_SiCl/NaI [19] were unsuccessful. Instead, **7** was obtained in good yield by the two-step sequence of oxidation employing ammonium cerium(IV) nitrate (CAN) [20] to afford the corresponding quinone (not shown), followed by reduction with sodium hydrosulfite [21]. With the nitrile in hand, we next attempted to reduce that functional group to an amine to which a leucine moiety could be appended. Unfortunately, reactions of **6** and **8** utilizing catalytic hydrogenation, lithium aluminium hydride by itself as well as with added aluminium chloride or samarium iodide produced only trace amounts of an amine (e.g., **9**). When samarium iodide was used as the reagent, the only discernible product (not fully characterized) appeared to be the saturated derivative of **6** (2H triplets at δ 2.61 and 2.92; *J* = 7.3 Hz in the ^1^H NMR spectrum; signal at 2245 cm^−1^ in the IR spectrum).

**Scheme 3:**
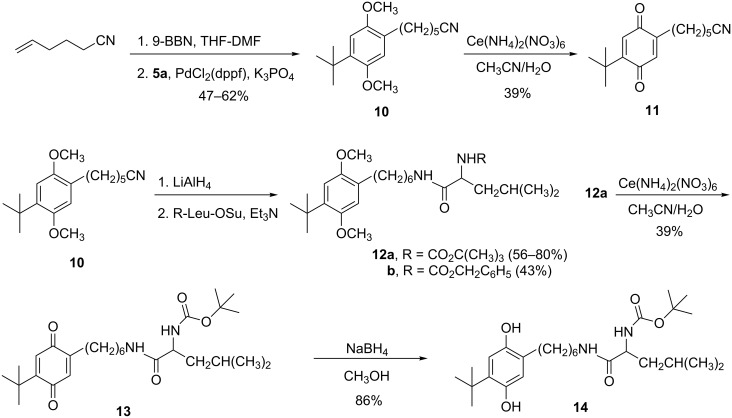
Suzuki approach to a tethered hydroquinone.

We next turned our attention to Suzuki coupling [10]. The reaction of commercially available 5-hexenenitrile with 9-BBN-H, followed by addition of bromide **5a**, potassium phosphate and PdCl_2_(dppf)_2_ in DMF at 85 °C for 3.5 h gave the coupled product **10** in 47–63% yields (Scheme 3). In contrast to the case of the Heck coupling products (**6** and **8**, Scheme 2), nitrile **10** underwent facile reduction with lithium aluminium hydride. The product amine was not isolated. Instead, it was immediately reacted with the *N*-hydroxysuccinimidyl ester of Boc-leucine to give **12a** in yields of 56–80% overall for two steps. We also obtained the corresponding *Z*-protected analogue **12b** in 43% overall yield for two steps. Both **10** and **12a** could be oxidized with CAN to the corresponding quinones **11** and **13**, respectively. Numerous unsuccessful attempts were made to convert the dimethoxy compound [12] or one of its precursors into a hydroquinone (e.g., **14**), either in one step (BBr_3_·S(CH_3_)_2_ [18] or (CH_3_)_3_SiCl/NaI [19]), or sequentially (CAN followed by Na_2_S_2_O_4_ or H_2_/Pd). What finally proved effective was the two step sequence of oxidation of **12a** with CAN followed by treatment of the resulting quinone **13** with sodium borohydride in dry methanol – a sequence that gave **14** (which upon standing readily re-oxidizes back to **13**) in 34% overall yield from **12a**.

A standard coupled ATPase activity assay showed that **14** was an active SERCA inhibitor with an IC_50_ value of 23 ± 7 μM (4 trials). This observation demonstrated that despite their considerable size in relation to the active BHQ entity, the tether and the protected leucine residue did not abolish inhibitory activity, although they caused an approximately 50-fold reduction in potency. This finding was significant because cleavage of a peptide carrier by PSA would result in a compound structurally similar to **14** (its deprotected version) that needs to be an active SERCA inhibitor to be of use against prostate cancer cells. Even though the Boc group of **14** would not be present in the final cleavage product, it has been demonstrated for TG-based inhibitors that its presence did not affect inhibitory potency [22]. In support of this observation, X-ray crystallography revealed the location of the Boc group in a solvent-exposed area on the surface of SERCA where it did not undergo major interactions with the enzyme [22]. Therefore, conducting the inhibition assay with the Boc-protected BHQ derivative facilitates a convenient direct comparison with inhibition results for TG-analogues that have been characterized with Boc groups present [23].

## Conclusion

Using Heck- and Suzuki-coupling reactions, we have developed a synthetic route that provides a BHQ template tethered to a leucine residue (**14**). Similar to its TG-based counterparts, **14** is an active SERCA inhibitor, which is a requirement for its potential use as an antiprostate cancer agent. To achieve the latter, the leucine residue will need to be extended to yield the full-length His-Ser-Ser-Lys-Leu-Gln-Leu peptide which can serve as a substrate for PSA. In future work, such a compound would need to be evaluated in living cells to assess its effects on cytosolic calcium levels and on the viability of both cancer and healthy cells.

## Supporting Information

File 1 Experimental.

File 2 NMR spectra.
