# Supplementary material for: Heck- and Suzuki-coupling approaches to novel hydroquinone inhibitors of calcium ATPase
Source: Beilstein J Org Chem. 2019 Apr 24;15:971–5. doi: 10.3762/bjoc.15.94 (PMC6541364; doi:10.3762/bjoc.15.94)
Supplement: File 1 — Experimental. [file Beilstein_J_Org_Chem-15-971-s001.pdf]

BEILSTEIN JOURNAL OF ORGANIC CHEMISTRY

Supporting Information

for

Heck- and Suzuki-coupling approaches to novel hydroquinone inhibitors of calcium ATPase

Robert J. Kempton, Taylor A. Kidd-Kautz, Soizic Laurenceau and Stefan Paula

Beilstein J. Org. Chem. **2019,** *15,* 971–975. doi:10.3762/bjoc.15.94

Experimental

License and Terms: This is a supporting information file under the terms of the Creative Commons Attribution License (http://creativecommons.org/licenses/by/4.0). Please note that the reuse, redistribution and reproduction in particular requires that the authors and source are credited.
The license is subject to the *Beilstein Journal of Organic Chemistry* terms and conditions: (https://www.beilstein-journals.org/bjoc)

Experimental

Organic Synthesis

Chemistry. Coupling reagents $PdCl_2$(dppf) and [(*t*-Bu)$_3$PH]BH_4 were purchased from Strem Chemicals (Newburyport, MA). All other reagents were purchased from Sigma-Aldrich (St. Louis, MO) or Fisher Scientific (Pittsburgh, PA) and were used without further purification. All reactions were carried out under an argon atmosphere. Melting points were measured with a Thomas Hoover melting point apparatus and are uncorrected. Thin layer chromatography (TLC) was performed using Baker-flex silica gel sheets and detection of spots was made by UV light and/or iodine vapours. Preparative chromatography was performed using either a CombiFlash Rf system (TELEDYNE ISCO) with silica gel cartridges, or on Analtech Uniplates TLC plates (silica gel GF; 20 × 20 cm; 2,000 μm). Proton (500 MHz) and carbon (125.7 MHz) NMR spectra were obtained on a JEOL Eclipse 500 MHz spectrometer. Chemical shifts are reported as parts per million (ppm) relative to TMS in $CDCl_3$ or acetone-d_6. For all compounds not previously characterized in the literature, high resolution mass spectra (HRMS) were obtained using either electron impact (EI) or electrospray (ESI) instruments at the Mass Spectrometry Laboratory at the University of Illinois at Urbana-Champaign, or with a ThermoFisher LTQArbitrap instrument in the Department of Chemistry at Purdue University.

1-Bromo-4-*tert*-butyl-2,5-dimethoxybenzene (5a). To a stirred solution of 2,5-dimethoxybromobenzene (**4a**, 2.06 g, 9.5 mmol) in *tert*-butyl chloride (10 mL) at 0 °C was added in portions ca. 0.5 g of aluminium chloride. The original pale yellow solution

turned dark yellow/green. Stirring was continued at 0 °C for 1.5 h. Work up consisted of adding ice to decompose excess aluminium chloride, followed by partitioning the reaction between ethyl acetate and water. The organic layer was washed with dil. aq $NaHCO_3$, water and dried over Na_2SO_4. Rotary evaporation gave a light orange oil that was partially purified by running it through a 2 cm pad of silica gel with 10% CH_2Cl_2 in hexane. The yield was 2.26 g (87%) of a colourless semi-crystalline solid that was used without further purification. An analytical sample was prepared by recrystallization from hexane. Mp 54-57 °C. (Lit: 57-58 °C [1]). ^{1}H-NMR ($CDCl_3$, 500 MHz): δ1.35 (s, 9H, Bu^t), 3.79 (s, 3H, OCH_3), 3.85 (s, 3H, OCH_3), 6.89 (s, 1H), 7.02 (s, 1H); ^{13}C-NMR ($CDCl_3$, 125 MHz): δ 29.6, 35.2, 55.9, 57.2, 108.7, 112.3, 116.9, 138.9, 149.7, 153.2.

2-Iodo-1,4-dimethoxybenzene (4b). Silfen was freshly prepared by thoroughly grinding $Fe(NO_3)_3(H_2O)_9$ (2.0 g) and silica gel (4.0 g; 230-400 mesh) in a mortar [2]. A 25 mL round-bottomed flask was charged with 600 mg of silfen, iodine (142 mg; 1.1 mmol), 1,4-dimethoxybenzene (138 mg; 1.0 mmol) and CH_2Cl_2 (2 mL), and the mixture was stirred at 25 °C overnight. The product was isolated by partitioning the reaction mixture between ethyl acetate and water, followed by washing the organic layer with 5% aq sodium thiosulfate and water, and drying (Na_2SO_4). Concentration by rotary evaporation gave an oil which was purified by CombiFlash chromatography (silica gel; 0–30% CH_2Cl_2 in hexane over 20 min) to afford a colourless oil (161 mg; 61%). ^{1}H-NMR ($CDCl_3$, 500 MHz): δ 3.74 (s, 3H, OCH_3), 3.81 (s, 3H, OCH_3), 6.74 (d, 1H, J = 8.8 Hz, H-6), 6.85 (dd, 1H , J = 8.8, 3.1 Hz, H-5), 7.30 (d, 1H, J = 3.1 Hz, H-3). ^{13}C-NMR ($CDCl_3$, 125 MHz): δ 56.0, 57.1, 86.2, 111.7, 114.7, 125.0, 152.7, 154.3.

1-Iodo-4-*tert*-butyl-2,5-dimethoxybenzene (5b). To a stirred solution of 2-iodo-1,4-dimethoxybenzene (**4b**, 264 mg; 1.0 mmol) and *tert*-butyl alcohol (0.25 mL) in glacial acetic acid (0.2 mL) at 0 °C was added dropwise over 2 min concentrated H_2SO_4 (0.3 mL). The reaction was stirred at 0 °C for 2 h and then allowed to warm to room temperature and stirred overnight. Work up consisted of partitioning the reaction mixture between water and ethyl acetate, followed by washing the organic layer with dil. aq $NaHCO_3$, water and drying (Na_2SO_4). Preparative TLC (hexane-CH_2Cl_2, 1:1) produced recovered starting material (67 mg) and the product (148 mg; 46%; 62% based on recovered starting material). Mp 79-82 °C (Lit: 81-83 °C [3]). ^{1}H-NMR ($CDCl_3$, 500 MHz): δ 1.35 (s, 9H, Bu^t), 3.79 (s, 3H, OCH_3), 3.83 (s, 3H, OCH_3), 6.81 (s, 1H), 7.20 (s, 1H).

1-Bromo-4-*tert*-amyl-2,5-dimethoxybenzene (5c). To a stirred solution of the bromide **4a** (583 mg; 2.7 mmol) in *tert*-amyl chloride (3 mL) at 0 °C was added in portions aluminium chloride (0.16 g). The mixture became bright yellow, then orange-red. Stirring was continued for 2 h at 0 °C. Work up was the same as for **5a**. Purification by preparative TLC (3:1 hexane-CH_2Cl_2) afforded 481 mg (62%) of a colourless oil. ^{1}H-NMR ($CDCl_3$, 500 MHz): δ 0.62 (t, 3H, *J* = 7.6 Hz, $CH_2\underline{CH_3}$), 1.31 (s, 6H), 1.80 (q, 2H, *J* = 7.6 Hz, $\underline{CH_2}CH_3$), 3.77 (s, 3H, OCH_3), 3.85 (s, 3H, OCH_3), 6.82 (s, 1H), 7.01 (s, 1H); ^{13}C-NMR ($CDCl_3$, 125 MHz): δ 9.6, 27.8, 33.1, 38.8, 56.0, 57.2, 108.7, 113.5, 116.7, 137.3, 149.7, 153.2. MS: M^+ at m/z 286/288; base peak at m/z 257/259. HRMS: calcd for $C_{13}H_{19}BrO_2$: 286.05684; found 286.05830.

1-Bromo-4-(1-methylcyclopentyl)-2,5-dimethoxybenzene (5d). A solution of the bromide **4a** (1.4 g; 6.5 mmol) and 1-methylcyclopentanol (1 g; 10.2 mmol) in glacial acetic acid (5 mL) was chilled to 0 °C and concd H_2SO_4 (5 mL) was added dropwise over 40 minutes. The reaction turned purple then orange. Stirring at 0 °C was continued overnight. Work up was the same as for **5a**. Purification by preparative TLC (hexane-CH_2Cl_2, 2:1) gave a colourless oil (540 mg; 28%). ^{1}H-NMR ($CDCl_3$, 500 MHz): δ 1.23 (s, 3H), 1.66-1.78 (m, 4H), 1.82-1.88 (m, 2H), 1.89-1.94 (m, 2H), 3.77 (s, 3H, OCH_3), 3.85 (s, 3H, OCH_3), 6.86 (s, 1H), 7.02 (s, 1H). ^{13}C-NMR ($CDCl_3$, 125 MHz): δ 23.5, 26.1, 38.8, 46.4, 56.0, 57.2, 108.4, 112.5, 116.6, 139.7, 149.7, 152.8. HRMS calcd for $C_{14}H_{20}BrO_2$ (MH^+) 299.0647; found 299.0655.

(*E*)-3-(2,5-Dimethoxyphenyl)acrylonitrile (6). A mixture of the bromide **4a** (450 mg; 2.1 mmol), *N*,*N*-dicyclohexylmethylamine (525 mg; 2.7 mmol), acrylonitrile (277 mg; 5.2 mmol), $Pd_2(dba)_3$ (94 mg; 4.9 mole % relative to **4a**), *tert*-Bu_3PHBF_4 (120 mg; 0.41 mmol) and dry dioxane (3 mL) were added to a 10 mL Schlenk flask and sealed. The reaction mixture was stirred for 2.5 h. in an oil bath heated to 110 °C. After cooling the reaction mixture was partitioned between ethyl acetate and water. The organic layer was washed with 5% aq. HCl and water and dried (Na_2SO_4). The crude product was partially purified by running through a 3 cm pad of silica gel using methylene chloride-hexane (1:1), affording 348 mg (89%) of a pale yellow solid. An analytical sample of the pure (*E*) isomer was obtained by recrystallization from hexane. Mp 71-73 °C. ^{1}H-NMR ($CDCl_3$, 500 MHz): δ 3.78 (s, 3H, OCH_3), 3.84 (s, 3H, OCH_3), 6.03 (d, 1H, J = 16.9 Hz,

H-2), 6.86 (d, 1H, *J* = 8.8 Hz, H-3'), 6.90 (d, 1H, *J* = 2.8 Hz, H-6'), 6.94 (dd, 1H, *J* = 2.8, 8.8 Hz, H-4'), 7.61 (d, 1H, *J* = 16.9 Hz, H-3). ^{13}C-NMR ($CDCl_3$, 125 MHz): δ 55.9, 56.1, 97.3, 112.6, 113.5, 117.8, 119.0, 123.1, 146.3, 152.7, 153.6. HRMS: calcd for $C_{11}H_{12}NO_2$ (MH^+): 190.0868; found: 190.0859.

(*E*)-3-(4-*tert*-Butyl-2,5-dimethoxyphenyl)acrylonitrile (8). A 10 mL Schlenk flask was charged with **5a** (186 mg; 0.68 mmol), *N*,*N*-dicyclohexylmethylamine (146 mg; 0.75 mmol), acrylonitrile (122 mg; 2.30 mmol), *tert*-Bu_3PHBF_4 (42 mg; 0.14 mmol), $Pd_2(dba)_3$ (31 mg; 0.034 mmol; 5 mole % relative to **5a**) and dry dioxane (1 mL). The sealed flask was stirred for 24 h in an oil bath heated to 120 °C. Work up and partial purification as was performed for **6** gave a yellow oil. Preparative TLC (hexane-methylene chloride, 2:1) of this oil gave two major bands. The higher R_f band contained 63 mg of recovered starting material **5a**; the lower band gave 76 mg (46%; 82% based on recovered starting material) of the desired product. Recrystallization from hexane gave white crystals. Mp 106-108 °C. ^{1}H-NMR ($CDCl_3$, 500 MHz): δ 1.37 (s, 9H, Bu^t), 3.82 (s, 3H, OCH_3), 3.86 (s, 3H, OCH_3), 6.01 (d, 1H, *J* = 17.0 Hz, H-2), 6.81 (s, 1H), 6.88 (s, 1H), 7.57 (d, 1H, *J* = 17.0 Hz, H-3). ^{13}C-NMR ($CDCl_3$, 125 MHz): δ 29.6, 35.6, 55.7, 56.1, 96.0, 111.1, 111.2, 119.3, 120.2, 143.8, 146.2, 152.5, 152.7. HRMS calcd for $C_{15}H_{19}NO_2$: 245.1416; found: 245.1418.

(*E*)-3-(2,5-Dihydroxyphenyl)acrylonitrile (7). To a rapidly stirred solution of **6** (104 mg; 0.55 mmol) in THF (3 mL) at room temperature was added a solution of ammonium cerium(IV) nitrate (CAN) (1.07 g; 1.95 mmol) in water (2 mL) dropwise over 6 min.

Stirring was continued for 30 min after the addition was complete. The dark orange solution was then partitioned between ethyl acetate and water. The organic layer was washed with water, brine and dried (Na_2SO_4), then concentrated to a dark orange solid. The solid was immediately taken up in ethyl acetate (6 mL) and stirred vigorously for 15 min with a freshly prepared solution of sodium hydrosulfite (700 mg; 4.0 mmol) in water (6 mL). The organic layer was washed with water and brine, dried and concentrated to a pale yellow solid. Purification was accomplished by preparative TLC (hexane-ethyl acetate, 2:1; two developments) to afford 50 mg (56%) of a pale yellow solid. Mp 160-163 °C. ^{1}H-NMR (acetone-d_6, 500 MHz): δ 6.26 (d, 1H, J = 16.8 Hz, H-2), 6.80 (m, 2H), 6.96 (d, 1H, J = 2.8 Hz, H-6'), 7.60 (d, J = 16.8 Hz, 1H, H-3). ^{13}C-NMR (acetone-d_6, 125 MHz): δ 96.3, 114.1, 117.2, 118.9, 119.7, 121.3, 146.2, 149.8, 150.6. HRMS: calcd for $C_9H_8NO_2$ (MH^+): 162.0555; found 162.0547.

6-(4-*tert*-Butyl-2,5-dimethoxyphenyl)hexanenitrile (10). A dry 25 mL round bottomed flask was charged with 5-hexenenitrile (200 mg; 2.1 mmol) and chilled to 0 °C. To this was added dropwise over 5 min 9-BBN-H (4.5 mL; 0.5 M in THF; 2.25 mmol). After the addition was complete the milky white solution was allowed to warm to room temperature and stirring was continued for 2 h. At the end of this period, K_3PO_4 (1.8 g; 8.4 mmol; 4 equiv) was added followed by a solution of the bromide **5a** (554 mg; 2.0 mmol) in dry DMF (10 mL), and finally $PdCl_2(dppf)_2$ (44 mg; 0.06 mmol; 3 mol % relative to **5a**). The reaction was heated at 65 °C for 2 days. Work up consisted of partitioning the reaction between ethyl acetate and water and washing the organic layer with water and brine. Drying (Na_2SO_4) and rotary evaporation resulted in a dark red oil. This oil

was partially purified by running it through a small pad of silica gel using CH_2Cl_2, yielding 490 mg of a pale yellow oil. Isolation of the product was accomplished using CombiFlash chromatography (silica gel; 100% hexane for 3 min; then 0–50% CH_2Cl_2 in hexane from 3–18 min; then 50–100% CH_2Cl_2 from 18-23 min). Starting bromide **5a** (196 mg) was recovered along with the product (218 mg; 37%; 57% based on recovered **5a**) as a pale yellow oil. ^{1}H-NMR ($CDCl_3$, 500 MHz): δ 1.36 (s, 9H, Bu^t), 1.51 (m, 2H), 1.61 (m, 2H), 1.70 (quint, 2H, J = 7.1 Hz), 2.34 (t, 2H, J = 7.1 Hz), 2.57 (t, 2H, J = 7.9 Hz), 3.79 (s, 3H, OCH_3), 3.80 (s, 3H, OCH_3), 6.66 (s, 1H), 6.81 (s, 1H). ^{13}C-NMR ($CDCl_3$, 125 MHz): δ 17.2, 25.4, 28.6, 29.3, 29.7, 29.9, 34.9, 56.0, 56.3, 110.6, 114.3, 120.0, 128.5, 136.8, 151.0, 152.5. HRMS: calcd for $C_{18}H_{27}NO_2$: 289.20418; found 289.20468.

6-(4-*tert*-Butyl-3,6-dioxocyclohexa-1,4-dien-1-yl)hexanenitrile (11). To a stirred solution of **10** (49 mg; 0.17 mmol) in acetonitrile (1 mL) at 0 °C was added dropwise over 1 min a chilled solution of ammonium cerium(IV) nitrate (CAN) (143 mg; 0.26 mmol). The reaction immediately turned green and eventually bright yellow. Stirring was continued at 0 °C for 45 min. Workup consisted of partitioning between ethyl acetate and water. Washing of the organic layer (H_2O, 2X), followed by drying (Na_2SO_4) and concentration gave an orange oil. Preparative TLC on this oil (3:1 CH_2Cl_2-hexane) isolated the product (17 mg; 39%) as a yellow oil. ^{1}H-NMR ($CDCl_3$, 500 MHz): δ 1.26 (s, 9H, Bu^t), 1.52 (m, 4H), 1.69 (quint, 2H, J = 7.3 Hz), 2.35 (t, 2H, J = 7.0 Hz), 2.39 (br t, 2H), 6.44 (br s, 1H), 6.56 (s, 1H). ^{13}C-NMR ($CDCl_3$, 125 MHz): δ 17.1, 25.2, 27.2, 28.1,

28.4, 29.3, 35.1, 119.6, 131.8, 134.8, 147.2, 156.0, 187.9, 188.6. HRMS calcd for $C_{16}H_{22}NO_2$ (MH^+): 260.1651; found 260.1650.

***tert*-Butyl (1-((6-(4-(*tert*-butyl)-2,5-dimethoxyphenyl)hexyl)amino)-4-methyl-1-oxopentan-2-yl)carbamate (12a).** A dry Schlenk flask was charged with 1.0 M (in ether) lithium aluminium hydride (LAH; 2.3 mL; 2.3 mmol). The flask was chilled to 0 °C and a solution of the nitrile **10** (154 mg; 0.53 mmol) in dry ether (2 mL) was added slowly over 3 min. After stirring for 1.5 h at 0 °C excess LAH was decomposed by dropwise addition of ethyl acetate. The reaction mixture was then partitioned between ethyl acetate and water, and the organic layer was washed with water and brine, dried (Na_2SO_4) and concentrated to yield a colourless oil (154 mg). This oil was immediately transferred to a pear shaped flask containing Boc-Leu-OSu (230 mg; 0.70 mmol) dissolved in CH_2Cl_2 (3 mL). The flask was cooled to 0 °C and Et_3N (30 drops via a Pasteur pipette) was added with stirring. The reaction was allowed to warm to room temperature and stirred overnight. Workup was affected by partitioning the reaction mixture between ethyl acetate and water, followed by washing the organic layer with dil. aq HCl, dil. aq $NaHCO_3$, water and brine. Drying followed by concentration on a rotary evaporator afforded a pale yellow oil (302 mg) that was purified by preparative TLC (2.5% CH_3OH in CH_2Cl_2). The yield of the colourless oil was 217 mg (80%). ^{1}H-NMR ($CDCl_3$, 500 MHz): δ 0.92 (m, 6H), 1.31-1.68 (m, 29H, including sharp singlets at 1.35 and 1.42), 2.53 (t, 2H, *J* = 7.8 Hz), 3.22 (m, 2H), 3.77 (s, 3H, OCH_3), 3.78 (s, 3H, OCH_3), 4.04 (m, 1H), 4.90 (m, 1H), 6.14 (m, 1H), 6.66 (s, 1H), 6.80 (s, 1H). ^{13}C-NMR ($CDCl_3$, 125 MHz): δ 22.2, 23.0, 24.9, 26.8, 28.4, 29.4, 29.6, 29.9, 30.1, 34.9, 39.6,

41.3, 53.2, 55.9, 56.4, 80.1, 110.7, 114.3, 129.2, 136.5, 151.0, 152.5, 155.8, 172.4. HRMS calcd for $C_{29}H_{51}N_2O_5$ (MH^+): 507.3798; found 507.3796.

Benzyl (1-((6-(4-(*tert*-butyl)-2,5-dimethoxyphenyl)hexyl)amino)-4-methyl-1-oxopentan-2-yl)carbamate (12b). A dry Schlenk flask was charged with 1.0 M lithium aluminium hydride (LAH; 1.2 mL; 1.2 mmol). The flask was chilled to 0 °C and a solution of the nitrile **10** (85 mg; 0.29 mmol) in dry ether (1 mL) was added slowly over 1 min. After stirring for 1.5 h at 0 °C, excess LAH was decomposed by dropwise addition of ethyl acetate. The reaction was then partitioned between ethyl acetate and water, and the organic layer was washed with water and brine, dried (Na_2SO_4) and concentrated to yield a colourless oil (75 mg). This oil was immediately transferred to a pear shaped flask containing *Z*-Leu-OSu (140 mg; 0.39 mmol) dissolved in CH_2Cl_2 (2 mL). The flask was cooled to 0 °C and Et_3N (15 drops via a Pasteur pipette) was added with stirring. The reaction was allowed to warm to room temperature and stirred overnight. Workup was affected by partitioning the reaction between ethyl acetate and water, followed by washing the organic layer with dil aq HCl, dil aq $NaHCO_3$, water and brine. Drying followed by concentration on a rotary evaporator afforded a pale yellow oil (121 mg) that was purified by preparative TLC (hexane – ethyl acetate, 1:1). The yield of the colourless oil was 69 mg (43%). ^{1}H-NMR ($CDCl_3$, 500 MHz): δ 0.92 (m, 6H, CH($\underline{CH_3}$)$_2$), 1.35-1.38 (m, 13H, including sharp singlet at 1.37), 1.47-1.66 (m, 7H), 2.55 (t, 2H, *J* = 7.5 Hz), 3.19 (m, 2H), 3.75 (s, 3H, OCH_3), 3.78 (s, 3H, OCH_3), 4.15 (m, 1H), 5.08 (m, 2H), 5.44 (d, 1H, *J* = 8.5 Hz), 6.26 (m, 1H), 6.67 (s, 1H), 6.81 (s, 1H), 7.29-7.33 (m, 5H). ^{13}C-NMR ($CDCl_3$, 125 MHz): δ 22.2, 23.0, 24.8, 26.8, 29.4, 29.5, 29.9, 30.1, 34.9, 39.7,

41.6, 53.7, 55.9, 56.4, 67.1, 110.7, 114.3, 128.1, 128.3, 128.6, 136.3, 136.5, 151.0, 152.5, 156.4, 172.2. HRMS: calcd for $C_{32}H_{48}N_2O_5$ + Na: 563.3461; found 563.3456.

***tert*-Butyl (1-((6-(4-(*tert*-butyl)-3,6-dioxocyclohexa-1,4-dien-1-yl)hexyl)amino)-4-methyl-1-oxopentan-2-yl)carbamate (13).** To a solution of **12a** (40 mg; 0.08 mmol) in acetonitrile (1 mL) chilled to 0 °C was added dropwise over 1 min a chilled solution of ammonium cerium(IV) nitrate (CAN) (85 mg; 0.15 mmol) in water (0.5 mL). Starting material was still present after stirring for 1 h so an additional amount of CAN (50 mg; 0.09 mmol) was added and stirring at 0 °C was continued for 7 h. The reaction was then partitioned between ethyl acetate and water and the organic layer was washed (H_2O), dried (Na_2SO_4) and concentrated to a light yellow oil. Purification was by preparative TLC (5% MeOH in CH_2Cl_2). The yield of the pale yellow oil was 15 mg (39%). ^{1}H-NMR ($CDCl_3$, 500 MHz): δ 0.93 (m, 6H), 1.27 (s, 9H, Bu^t), 1.35 (m, 6H), 1.43 (s, 9H, Bu^t), 1.48 (m, 4H), 1.67 (m, 1H), 2.34 (t, 2H, *J* = 7.0 Hz), 3.23 (m, 2H), 4.03 (m, 1H), 4.84 (m, 1H), 6.05 (m, 1H), 6.44 (s, 1H), 6.55 (s, 1H). ^{13}C-NMR ($CDCl_3$, 125 MHz): δ 14.3, 24.9, 26.5, 27.8, 28.2, 28.4, 29.0, 29.3, 29.4, 35.1, 39.4, 53.2, 60.5, 80.1, 131.8, 134.6, 147.7, 155.8, 155.9, 172.6, 188.1, 188.7. HRMS calcd for $C_{27}H_{45}N_2O_5$ (MH^+): 477.3328; found 477.3330.

***tert*-Butyl (1-((6-(4-(*tert*-butyl)-2,5-dihydroxyphenyl)hexyl)amino)-4-methyl-1-oxopentan-2-yl)carbamate (14)**. A stirred solution of **13** (53 mg; 0.12 mmol) in dry methanol (8 mL) was treated with $NaBH_4$ (27 mg; 0.71 mmol; 6.8 equiv) in one portion at room temperature. The reaction immediately turned from light yellow to colourless.

After stirring for 1.5 h, an additional portion of $NaBH_4$ (15 mg) was added. The reaction was quenched after 2 h by the slow addition of ice and then partitioned between water and ethyl acetate. The organic layer was washed with brine, dried and concentrated to yield a viscous oil (46 mg; 86%) of a product that showed one spot on TLC (hexane-EtOAc 1:1). ^{1}H-NMR ($CDCl_3$, 500 MHz): δ 0.91 (m, 6H, CH($\underline{CH_3}$)$_2$), 1.20-1.68 (m, 31H; including sharp singlets at 1.36 and 1.42), 2.47 (m, 2H), 3.19 (m, 2H), 5.18 (m, 1H), 6.36 (br s, 1H), 6.50 (s, 1H), 6.73 (s, 1H). ^{13}C-NMR ($CDCl_3$, 125 MHz): δ 14.3, 22.3, 22.8, 24.8, 25.8, 28.0, 28.4, 28.8, 29.1, 29.7, 34.4, 39.3, 41.3, 53.2, 60.6, 80.5, 114.4, 117.9, 126.4, 134.6, 146.8, 148.4, 156.2, 173.0. HRMS calcd for $C_{27}H_{47}N_2O_5$ (MH^+): 479.3485; found: 479.3476.

SERCA activity inhibition assay. SERCA was prepared in the form of microsomes from rabbit skeletal muscle tissue as described previously. The activity of the enzyme at varying concentrations of **14** was determined in an ATPase activity assay using the PK/LDH coupling system [4,5].

References

1. Hewgill, F. R.; Legge, F.; Webb, R. J. *Aust. J. Chem.,* **1993**, *46*, 1605-1611.

2. Beletskaya, I. P.; Cheprakov, A. V. *Chem. Rev.,* **2000**, *100*, 3009-3066.

3. Hayashi, N., Univ. of Toyama, Toyama, Japan, personal communication. The authors wish to thank Professor Hayashi for a generous sample of **5b**.

4. Paula, S.; Abell, J.; Deye, J.; Elam, C.; Lape, M.; Purnell, J.; Ratliff, R.; Zultowsky, J.; Kempton, R. J. *Bioorg. Med. Chem.*, **2009**, *17*, 6613-6619.

5. Lape, M.; Elam, C.; Versluis, M.; Kempton, R.; Paula, S. *Proteins,* **2008**, *70*, 639-649.
