# Supplementary material for: Heck- and Suzuki-coupling approaches to novel hydroquinone inhibitors of calcium ATPase
Source: Beilstein J Org Chem. 2019 Apr 24;15:971–5. doi: 10.3762/bjoc.15.94 (PMC6541364; doi:10.3762/bjoc.15.94)
Supplement: File 2 — NMR spectra. [file Beilstein_J_Org_Chem-15-971-s002.pdf]

BEILSTEIN JOURNAL OF ORGANIC CHEMISTRY

Supporting Information

for

Heck- and Suzuki-coupling approaches to novel hydroquinone inhibitors of calcium ATPase

Robert J. Kempton, Taylor A. Kidd-Kautz, Soizic Laurenceau and Stefan Paula

Beilstein J. Org. Chem. **2019,** *15,* 971–975. doi:10.3762/bjoc.15.94

NMR spectra

License and Terms: This is a supporting information file under the terms of the Creative Commons Attribution License (http://creativecommons.org/licenses/by/4.0). Please note that the reuse, redistribution and reproduction in particular requires that the authors and source are credited.
The license is subject to the *Beilstein Journal of Organic Chemistry* terms and conditions: (https://www.beilstein-journals.org/bjoc)

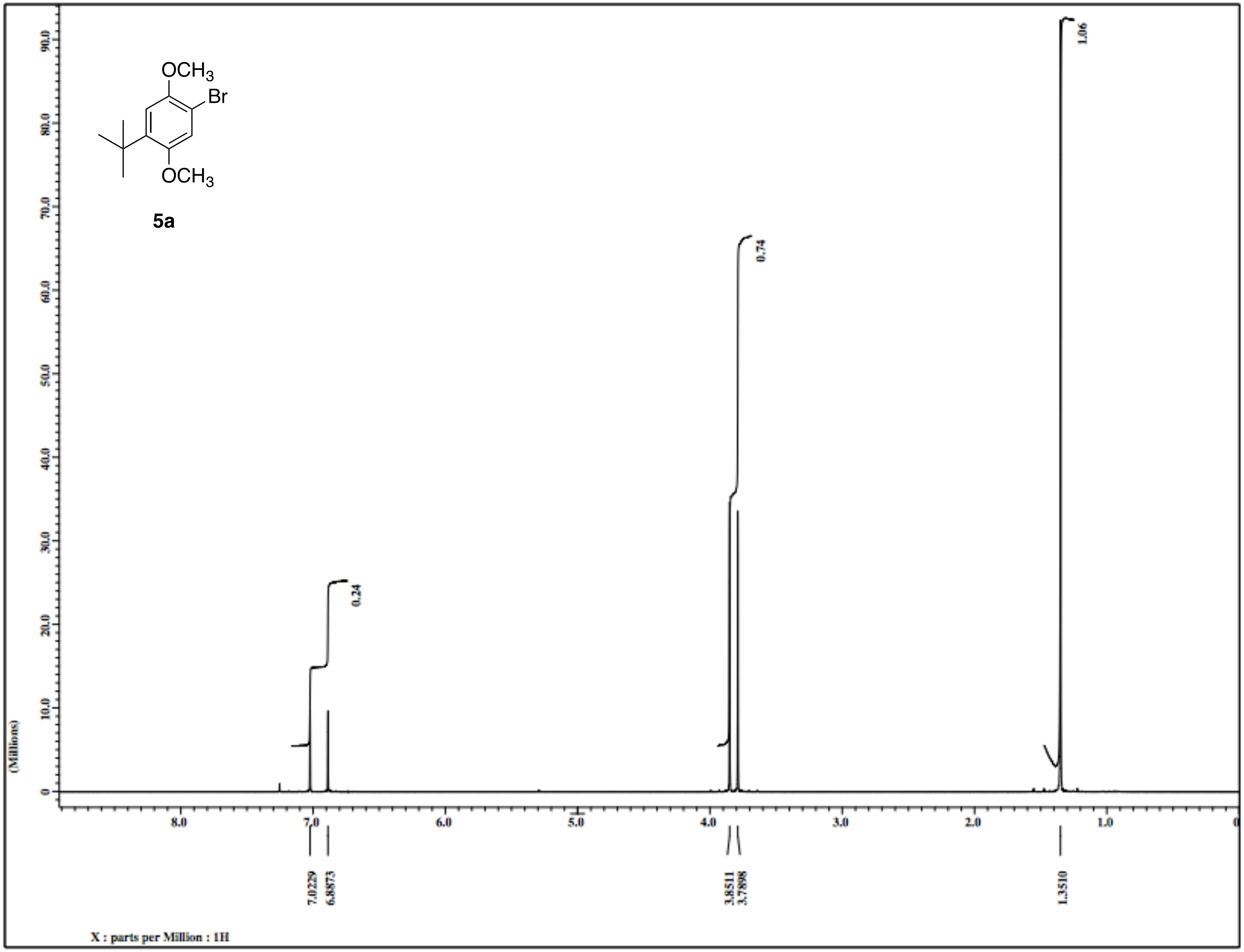
OCH3
Br
OCH3
5a
1.06
0.74
0.24
(Millions)
7.0229
6.8873
3.8511
3.7898
1.3510
X : parts per Million : 1H

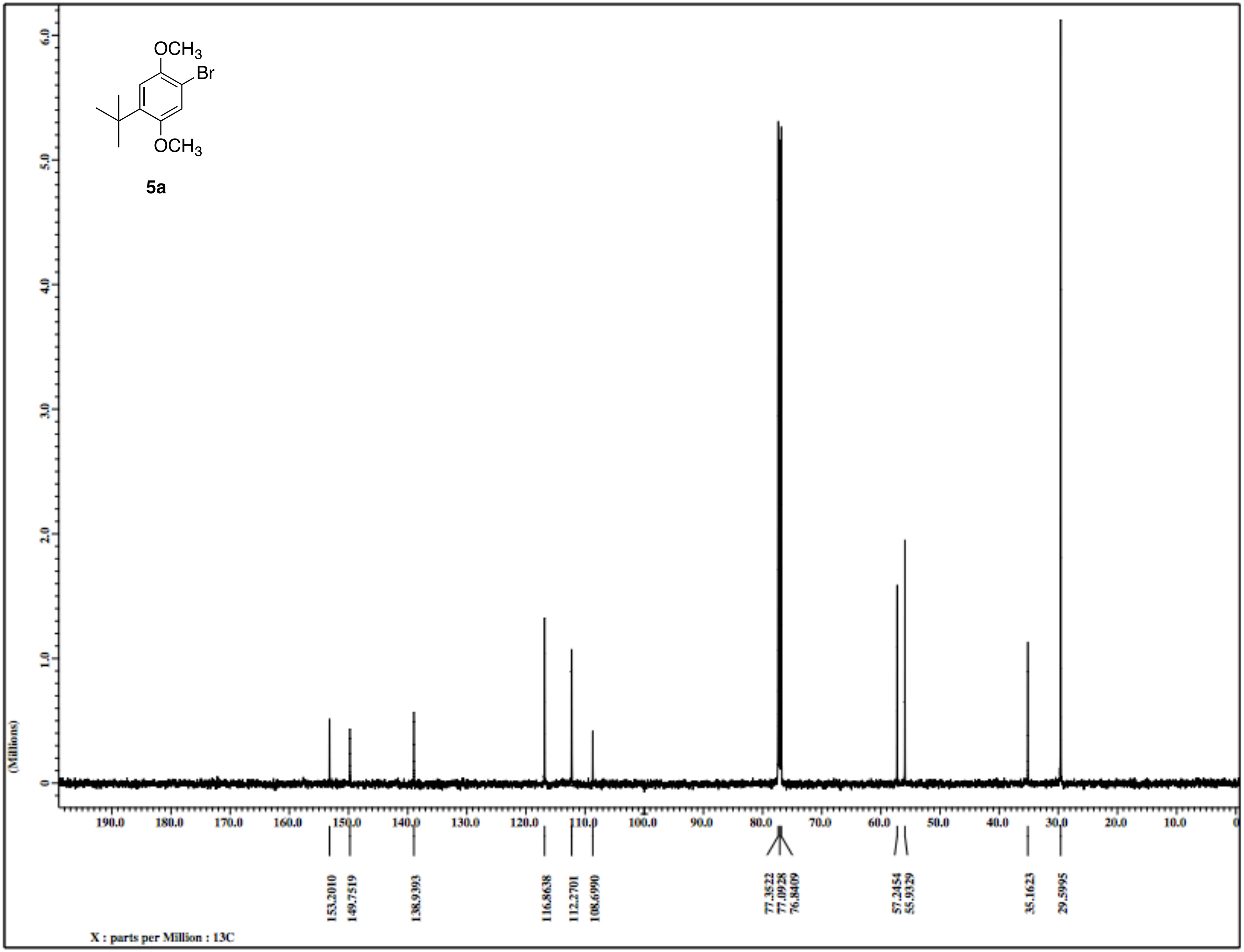

OCH3
Br
OCH3
5a
6.0
5.0
4.0
3.0
2.0
1.0
0
(Millions)
190.0
180.0
170.0
160.0
150.0
140.0
130.0
120.0
110.0
100.0
90.0
80.0
70.0
60.0
50.0
40.0
30.0
20.0
10.0
0
153.2010
149.7519
138.9393
116.8638
112.2701
108.6990
77.3522
77.0928
76.8409
57.2454
55.9329
35.1623
29.5995
X : parts per Million : 13C

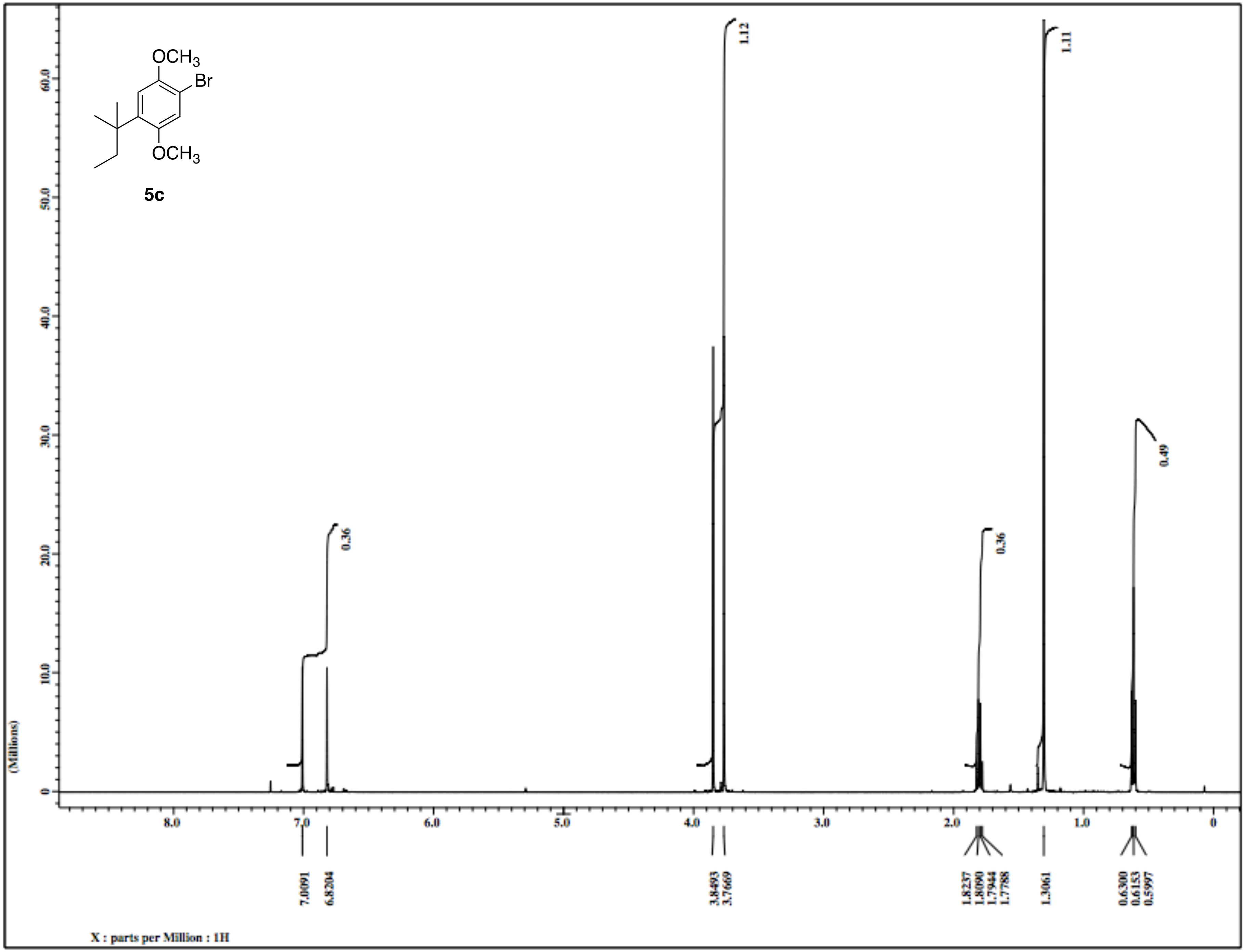
OCH3
Br
OCH3
5c
1.12
1.11
0.49
0.36
0.36
60.0
50.0
40.0
30.0
20.0
10.0
0
(Millions)
8.0
7.0
6.0
5.0
4.0
3.0
2.0
1.0
0
7.0091
6.8204
3.8493
3.7669
1.8237
1.8090
1.7944
1.7788
1.3061
0.6300
0.6153
0.5997
X : parts per Million : 1H

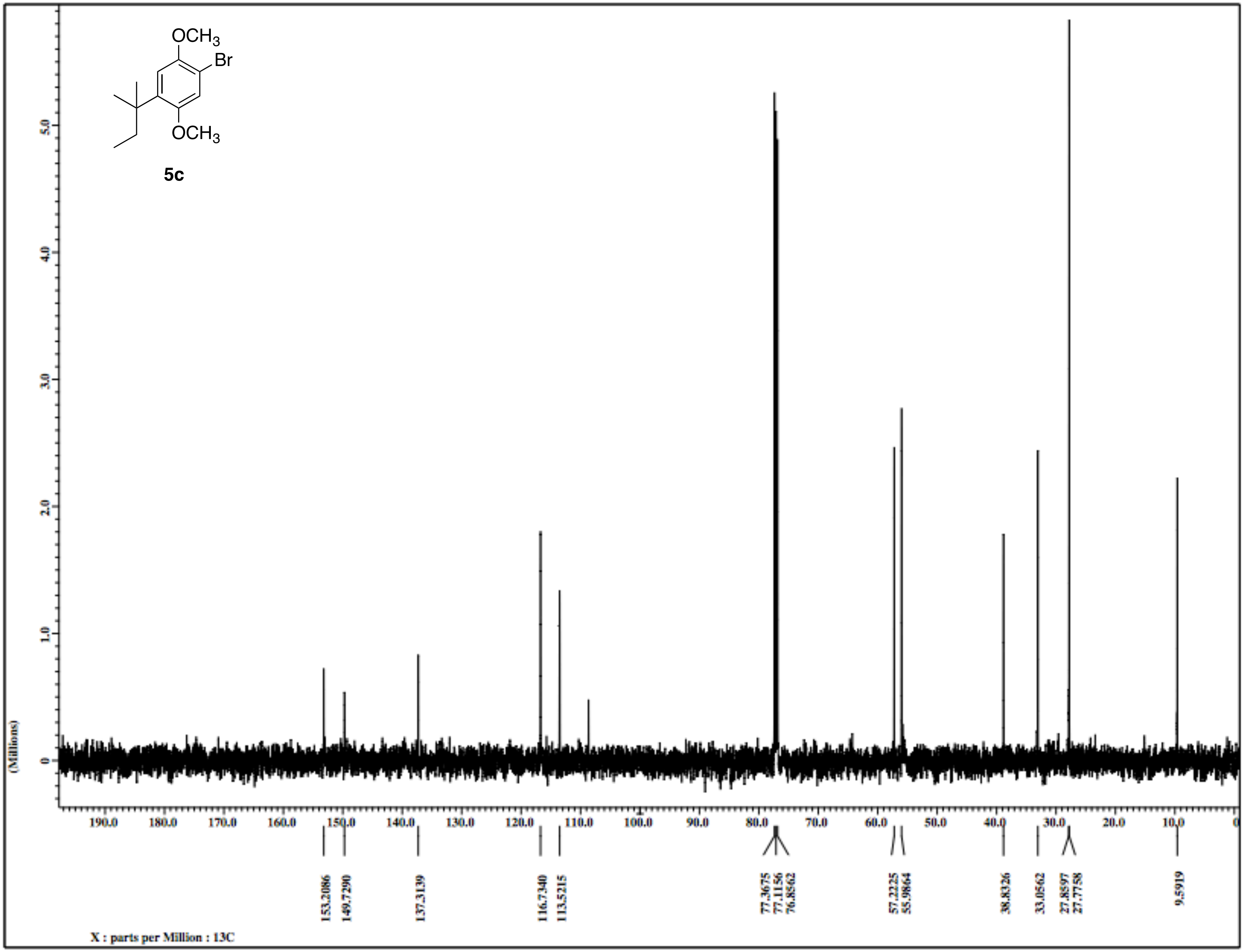

OCH3
Br
OCH3
5c
(Millions)
X : parts per Million : 13C
153.2086
149.7290
137.3139
116.7340
113.5215
77.3675
77.1156
76.8562
57.2225
55.9864
38.8326
33.0562
27.8597
27.7758
9.5919

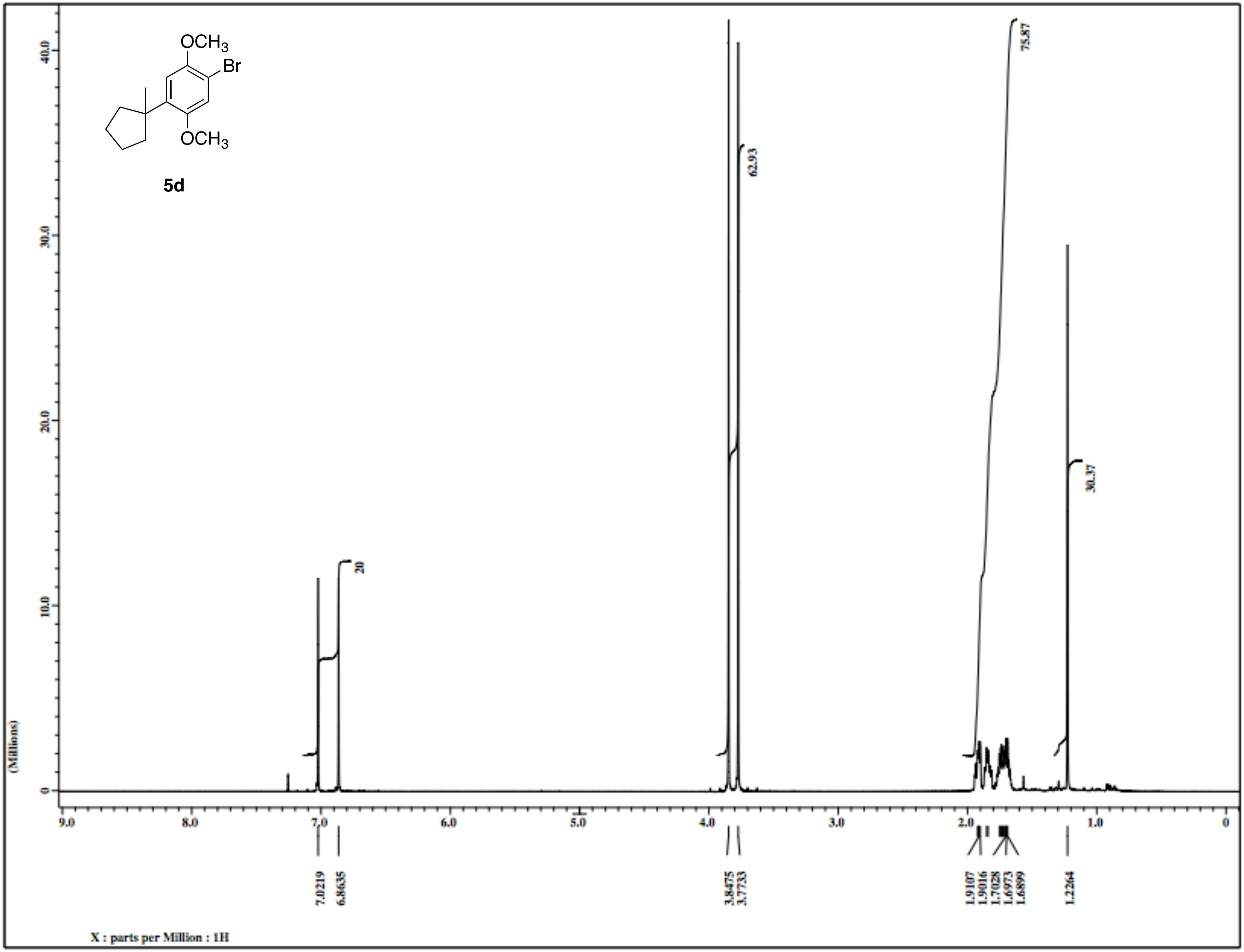
OCH3
Br
OCH3
5d
(Millions)
0
10.0
20.0
30.0
40.0
20
62.93
75.87
30.37
9.0
8.0
7.0
6.0
5.0
4.0
3.0
2.0
1.0
0
7.0219
6.8635
3.8475
3.7733
1.9107
1.9016
1.7028
1.6973
1.6899
1.2264
X : parts per Million : 1H

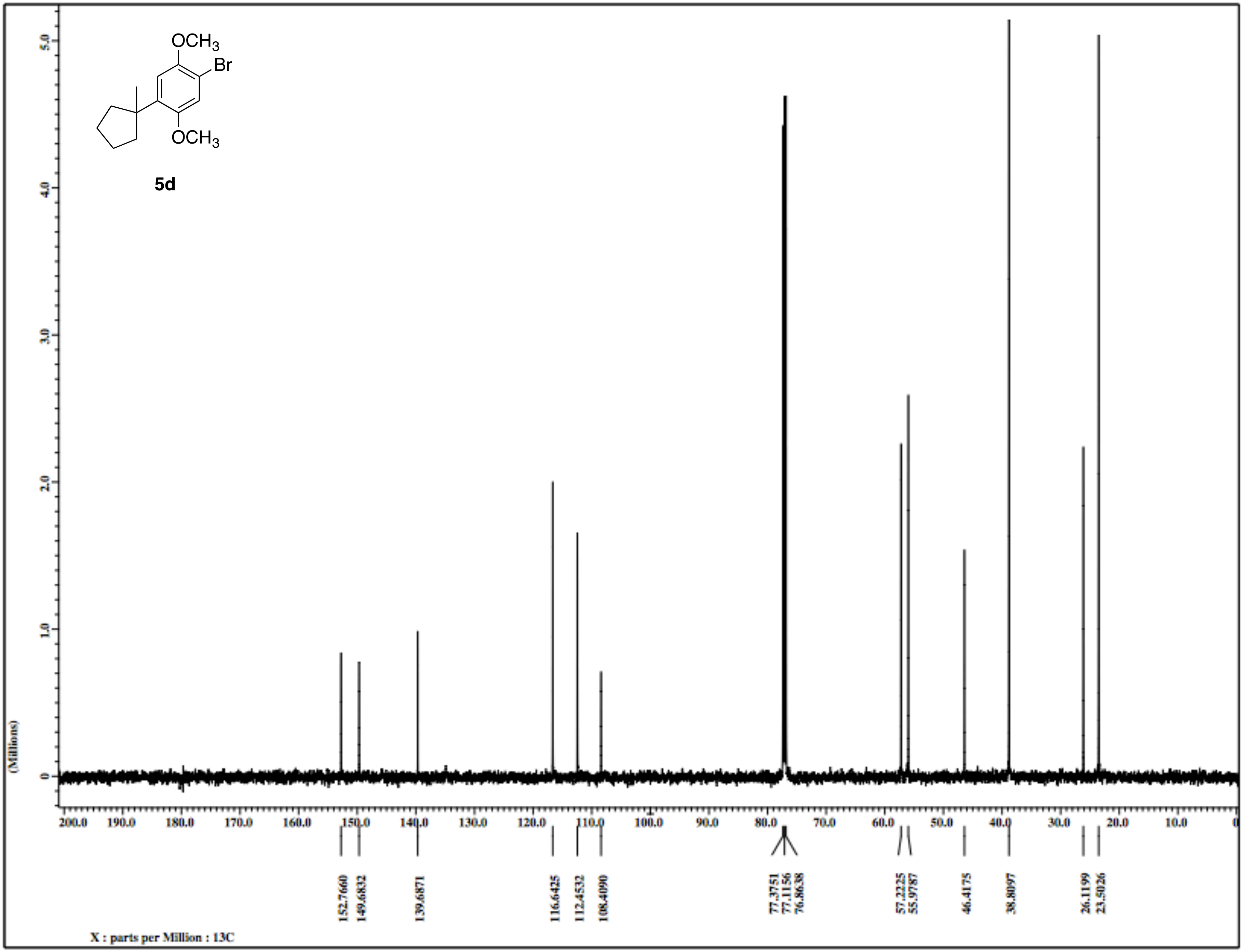
OCH3
Br
OCH3
5d
(Millions)
X : parts per Million : 13C
152.7660
149.6832
139.6871
116.6425
112.4532
108.4090
77.3751
77.1156
76.8638
57.2225
55.9787
46.4175
38.8097
26.1199
23.5026

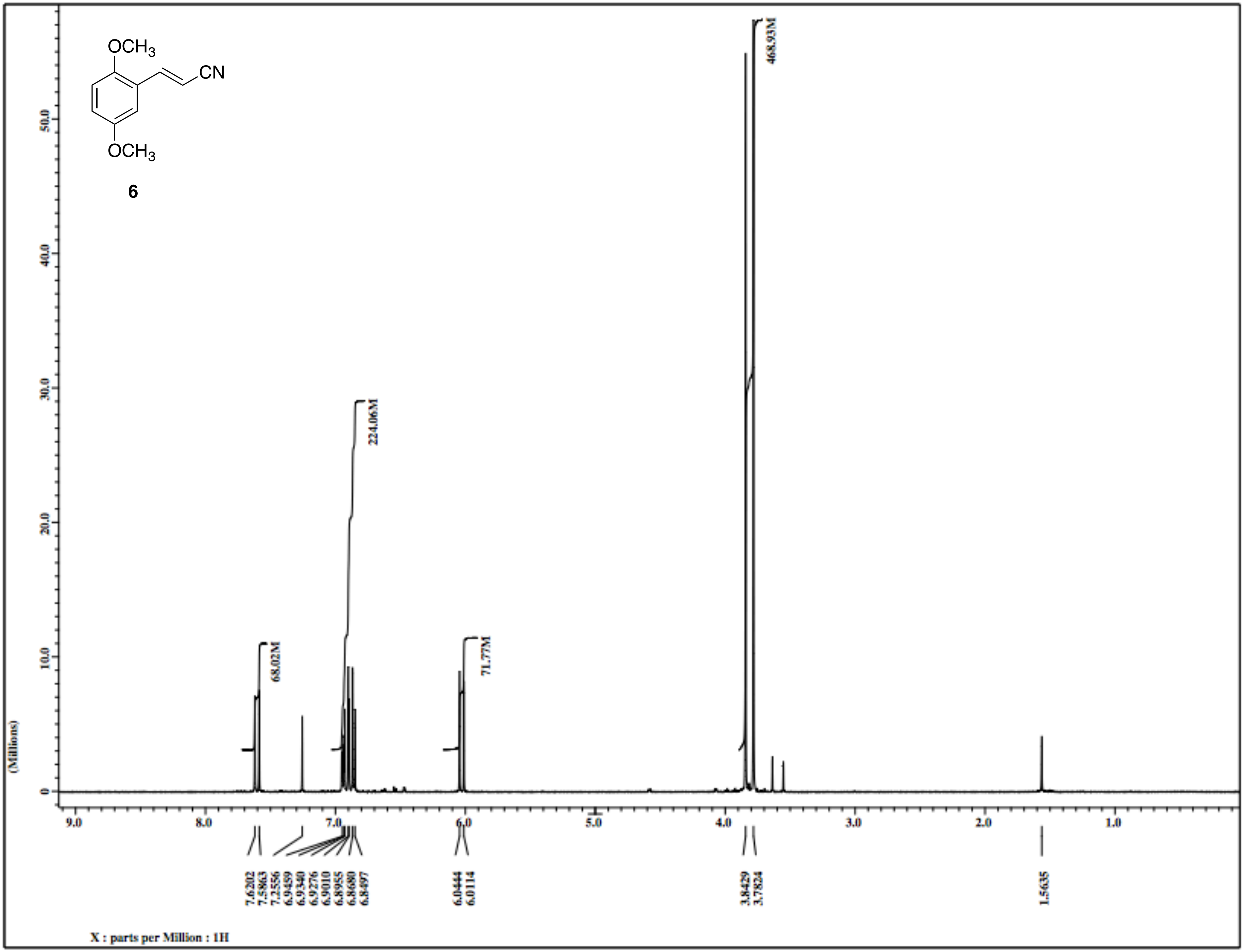

OCH3
CN
OCH3
6
(Millions)
0
10.0
20.0
30.0
40.0
50.0
68.02M
224.06M
71.77M
468.93M
9.0
8.0
7.0
6.0
5.0
4.0
3.0
2.0
1.0
7.6202
7.5863
7.2556
6.9459
6.9340
6.9276
6.9010
6.8955
6.8680
6.8497
6.0444
6.0114
3.8429
3.7824
1.5635
X : parts per Million : 1H

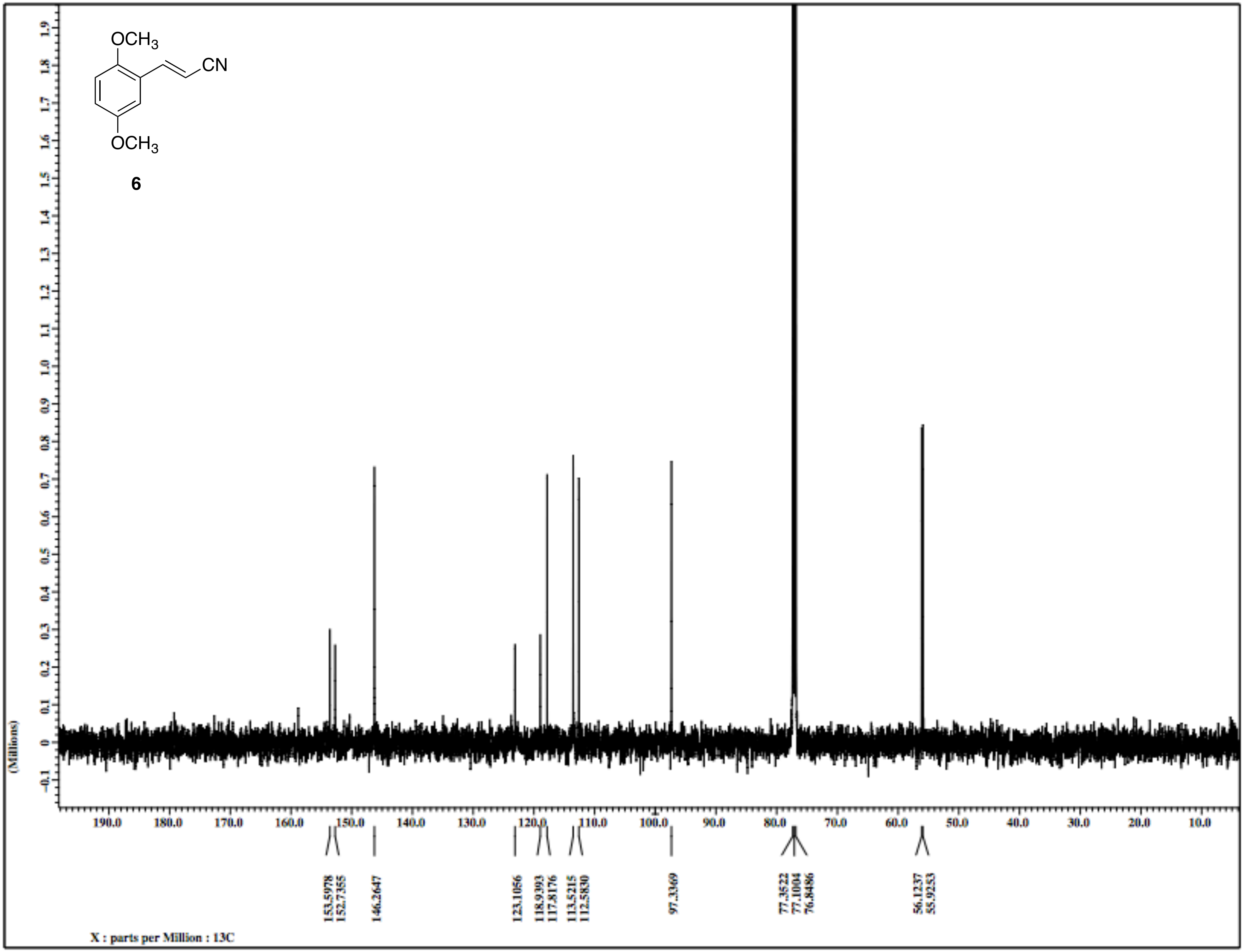

OCH3
CN
OCH3
6
(Millions)
190.0 180.0 170.0 160.0 150.0 140.0 130.0 120.0 110.0 100.0 90.0 80.0 70.0 60.0 50.0 40.0 30.0 20.0 10.0
153.5978
152.7355
146.2647
123.1056
118.9393
117.8176
113.5215
112.5830
97.3369
77.3522
77.1004
76.8486
56.1237
55.9253
X : parts per Million : 13C

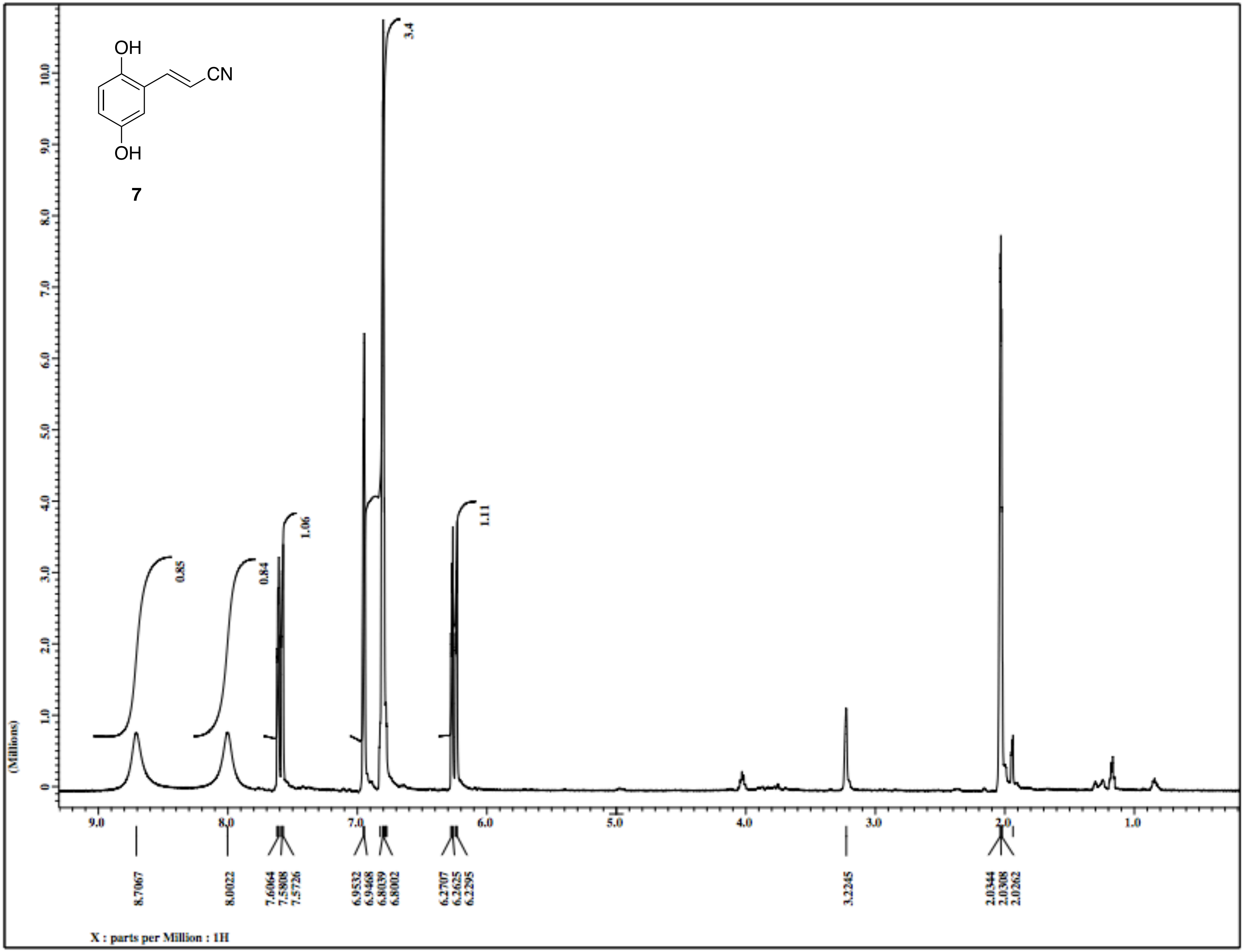
OH
CN
OH
7
(Millions)
3.4
1.06
0.85
0.84
1.11
8.7067
8.0022
7.6064
7.5808
7.5726
6.9532
6.9468
6.8039
6.8002
6.2707
6.2625
6.2295
3.2245
2.0344
2.0308
2.0262
X : parts per Million : 1H

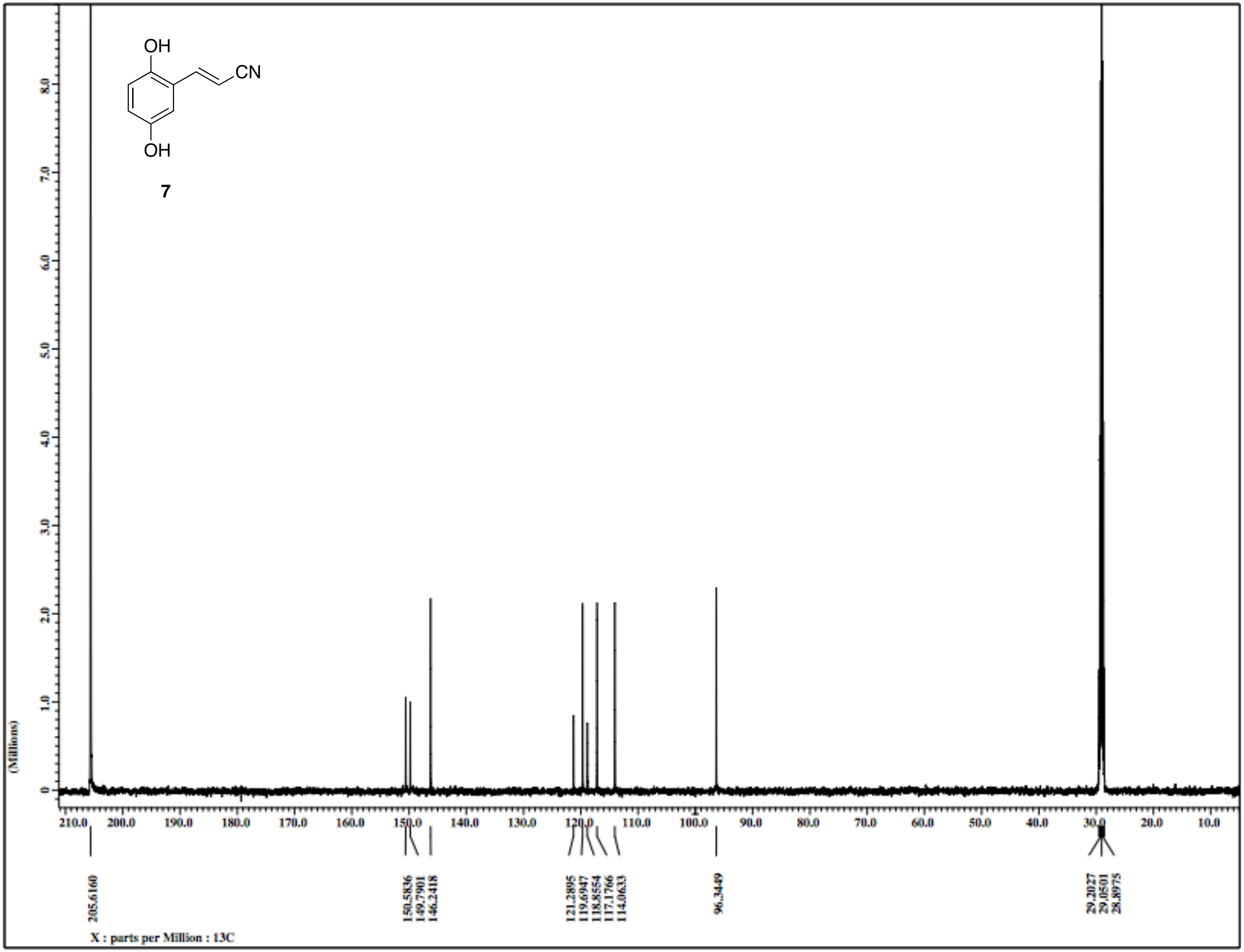

OH
CN
OH
7
(Millions)
0
1.0
2.0
3.0
4.0
5.0
6.0
7.0
8.0
210.0
200.0
190.0
180.0
170.0
160.0
150.0
140.0
130.0
120.0
110.0
100.0
90.0
80.0
70.0
60.0
50.0
40.0
30.0
20.0
10.0
205.6160
150.5836
149.7901
146.2418
121.2895
119.6947
118.8554
117.1766
114.0633
96.3449
29.2027
29.0501
28.8975
X : parts per Million : 13C

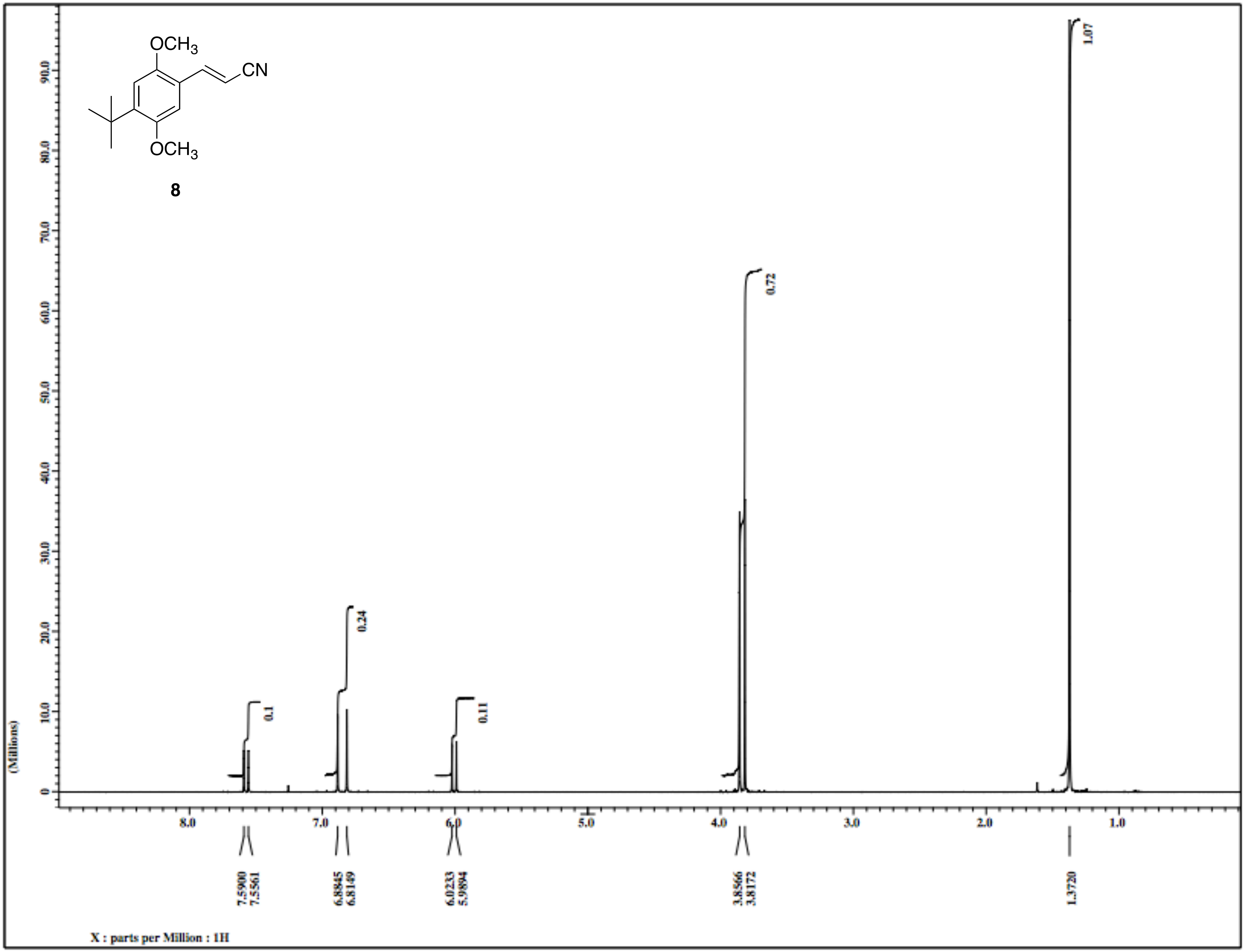

OCH3
CN
OCH3
8
(Millions)
0
10.0
20.0
30.0
40.0
50.0
60.0
70.0
80.0
90.0
0.1
0.24
0.11
0.72
1.07
8.0
7.0
6.0
5.0
4.0
3.0
2.0
1.0
7.5900
7.5561
6.8845
6.8149
6.0233
5.9894
3.8566
3.8172
1.3720
X : parts per Million : 1H

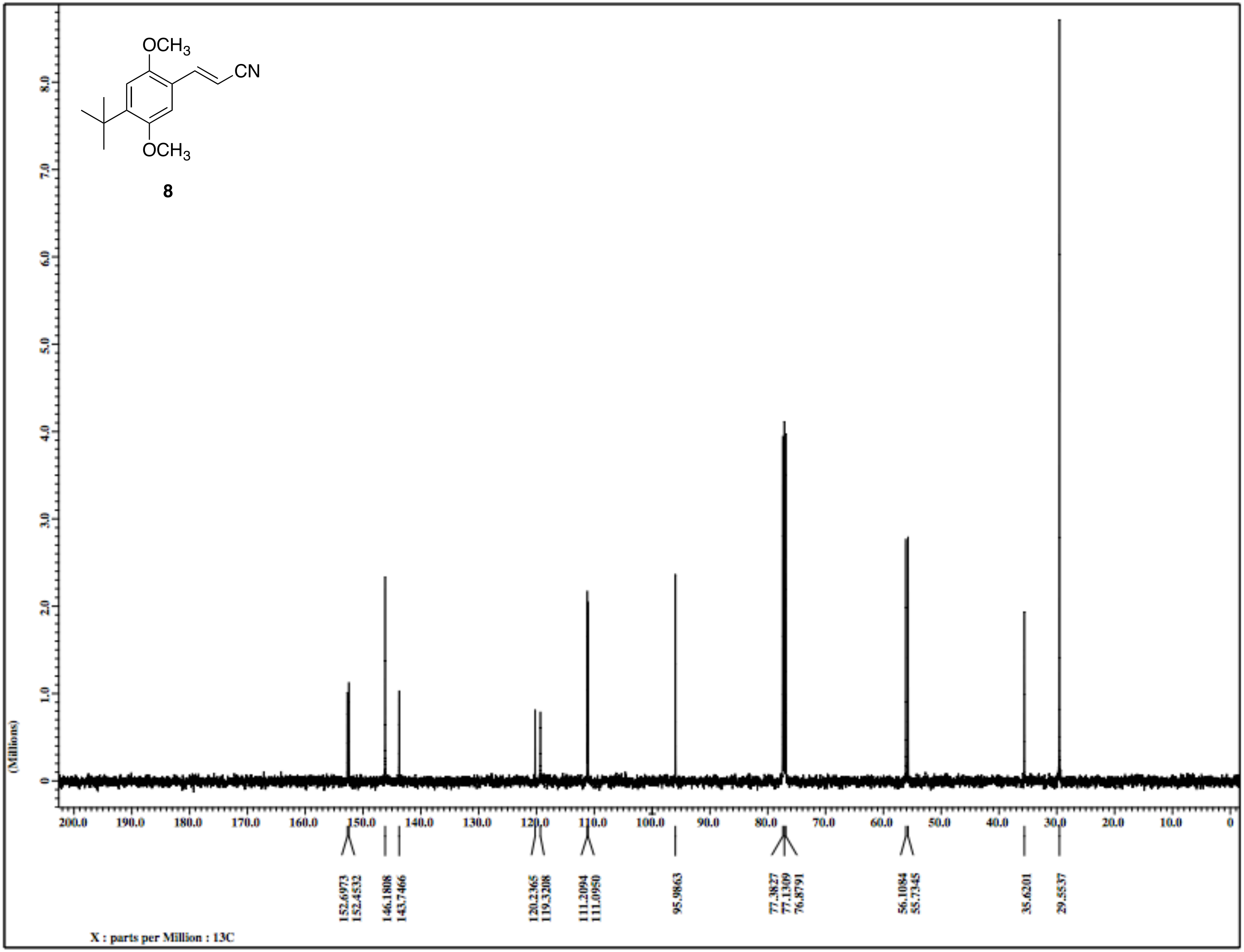

OCH3
CN
OCH3
8
(Millions)
X : parts per Million : 13C
152.6973
152.4532
146.1808
143.7466
120.2365
119.3208
111.2094
111.0950
95.9863
77.3827
77.1309
76.8791
56.1084
55.7345
35.6201
29.5537

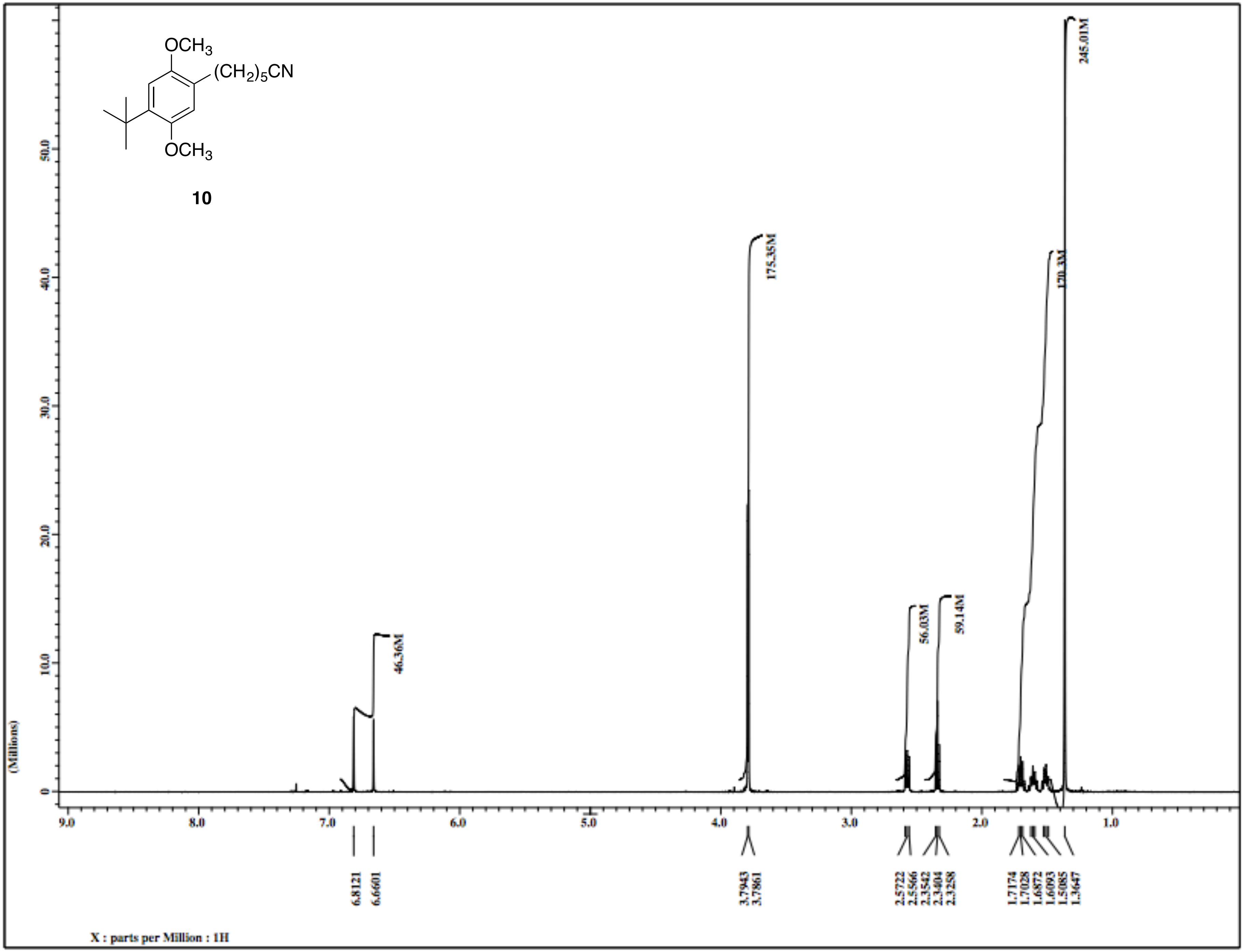

OCH3
(CH2)5CN
OCH3
10
245.01M
175.35M
170.3M
46.36M
56.03M
59.14M
(Millions)
0
10.0
20.0
30.0
40.0
50.0
9.0
8.0
7.0
6.0
5.0
4.0
3.0
2.0
1.0
6.8121
6.6601
3.7943
3.7861
2.5722
2.5566
2.3542
2.3404
2.3258
1.7174
1.7028
1.6872
1.6093
1.5085
1.3647
X : parts per Million : 1H

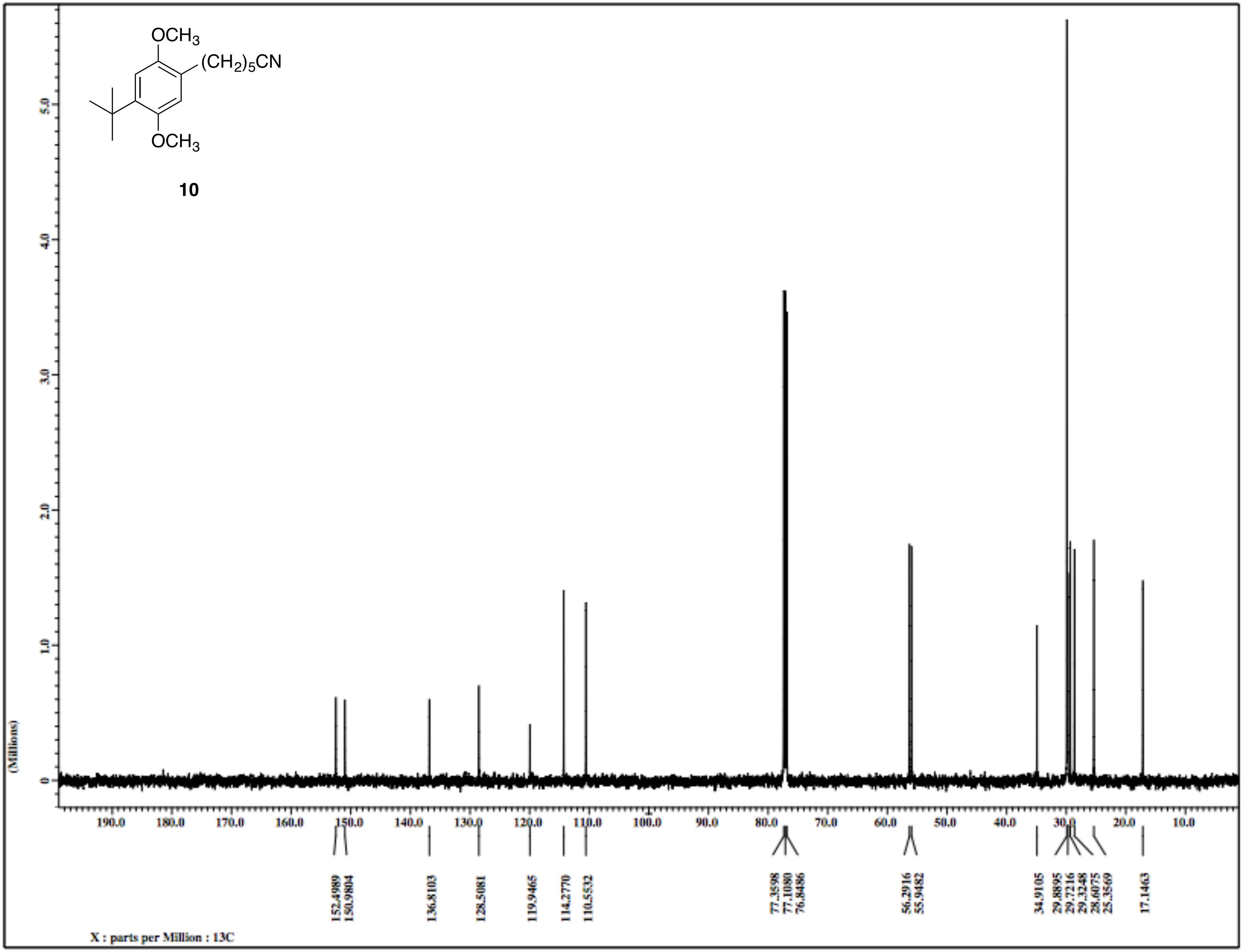

OCH3
(CH2)5CN
OCH3
10
(Millions)
5.0
4.0
3.0
2.0
1.0
0
190.0
180.0
170.0
160.0
150.0
140.0
130.0
120.0
110.0
100.0
90.0
80.0
70.0
60.0
50.0
40.0
30.0
20.0
10.0
152.4989
150.9804
136.8103
128.5081
119.9465
114.2770
110.5532
77.3598
77.1080
76.8486
56.2916
55.9482
34.9105
29.8895
29.7216
29.3248
28.6075
25.3569
17.1463
X : parts per Million : 13C

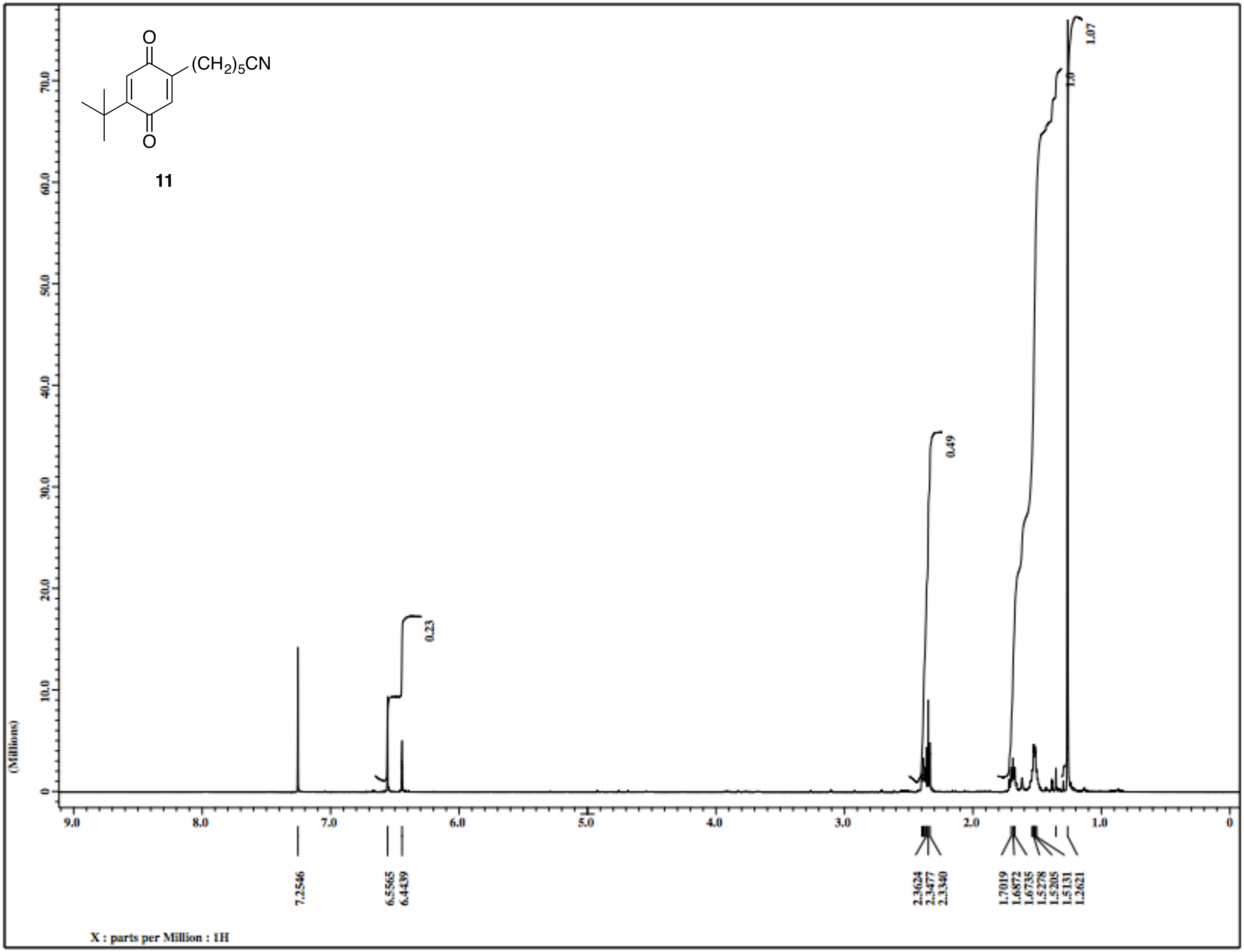

(CH2)5CN
11
70.0
60.0
50.0
40.0
30.0
20.0
10.0
0
(Millions)
1.07
1.0
0.49
0.23
9.0
8.0
7.0
6.0
5.0
4.0
3.0
2.0
1.0
0
7.2546
6.5565
6.4439
2.3624
2.3477
2.3340
1.7019
1.6872
1.6735
1.5278
1.5205
1.5131
1.2621
X : parts per Million : 1H

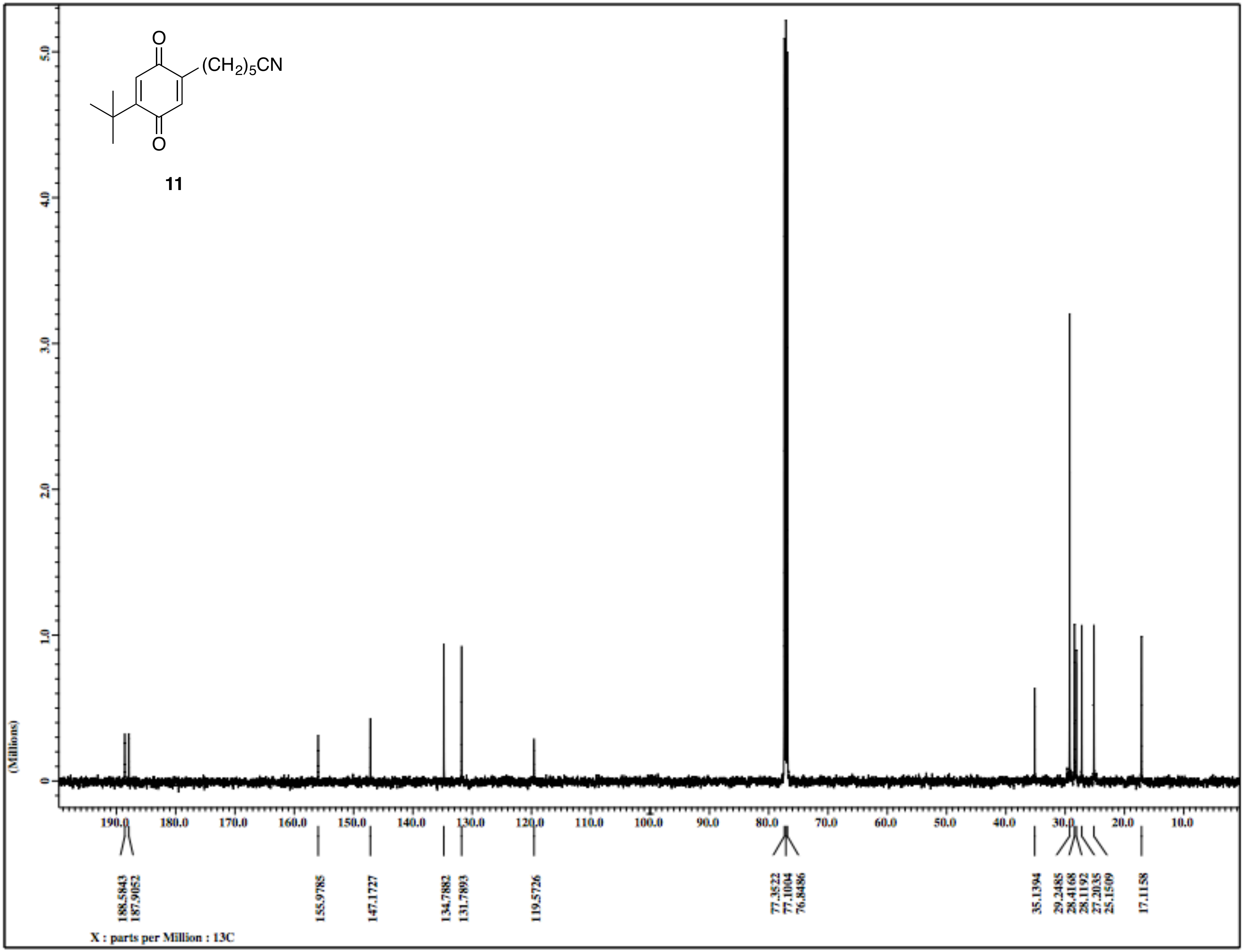

(CH2)5CN
O
O
11
(Millions)
X : parts per Million : 13C
188.5843
187.9052
155.9785
147.1727
134.7882
131.7893
119.5726
77.3522
77.1004
76.8486
35.1394
29.2485
28.4168
28.1192
27.2035
25.1509
17.1158

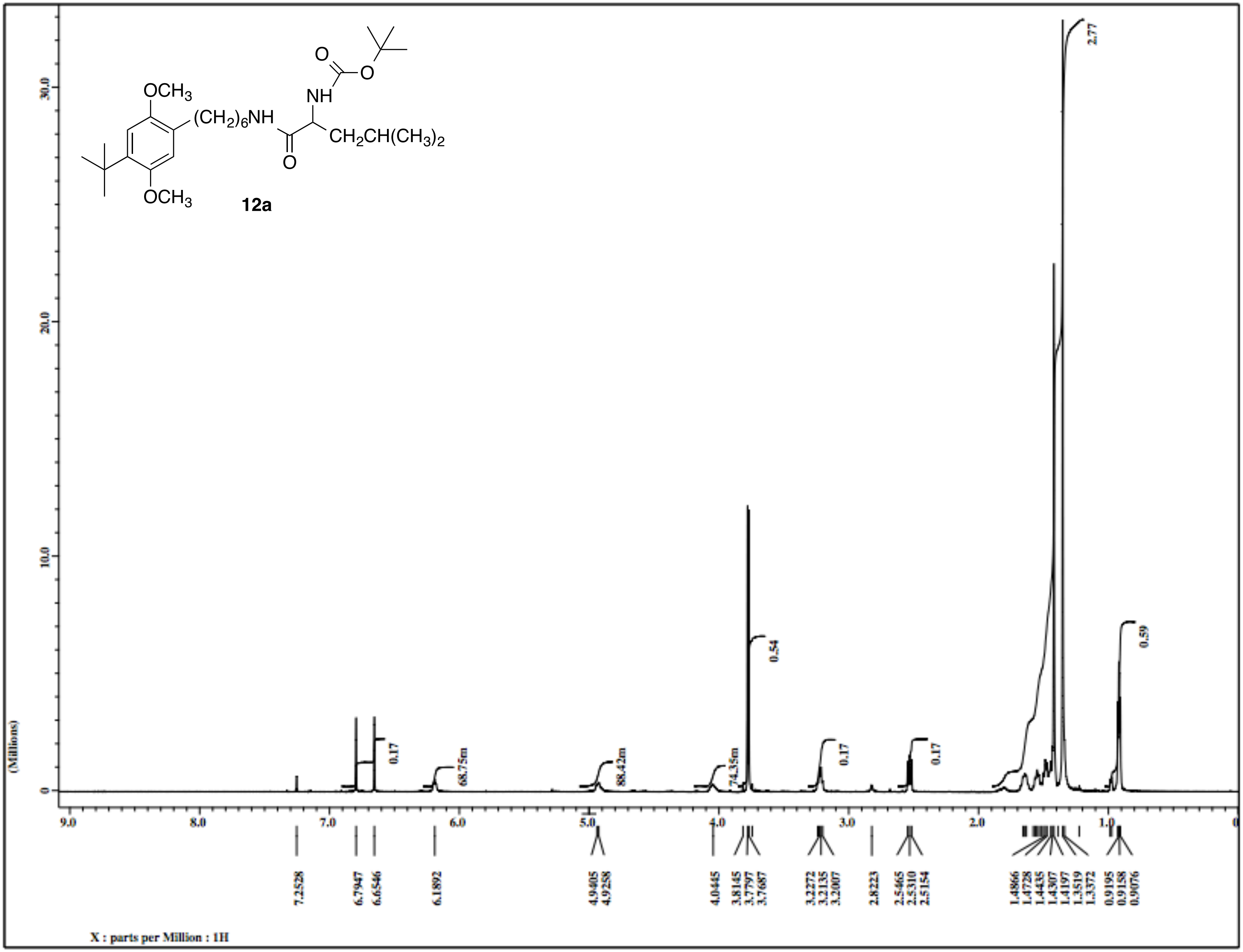

OCH3
(CH2)6NH
NH
O
O
CH2CH(CH3)2
OCH3
12a
(Millions)
30.0
20.0
10.0
0
0.17
68.75m
88.42m
74.35m
0.54
0.17
0.17
0.59
2.77
9.0 8.0 7.0 6.0 5.0 4.0 3.0 2.0 1.0 0
7.2528
6.7947
6.6546
6.1892
4.9405
4.9258
4.0445
3.8145
3.7797
3.7687
3.2272
3.2135
3.2007
2.8223
2.5465
2.5310
2.5154
1.4866
1.4728
1.4435
1.4307
1.4197
1.3519
1.3372
0.9195
0.9158
0.9076
X : parts per Million : 1H

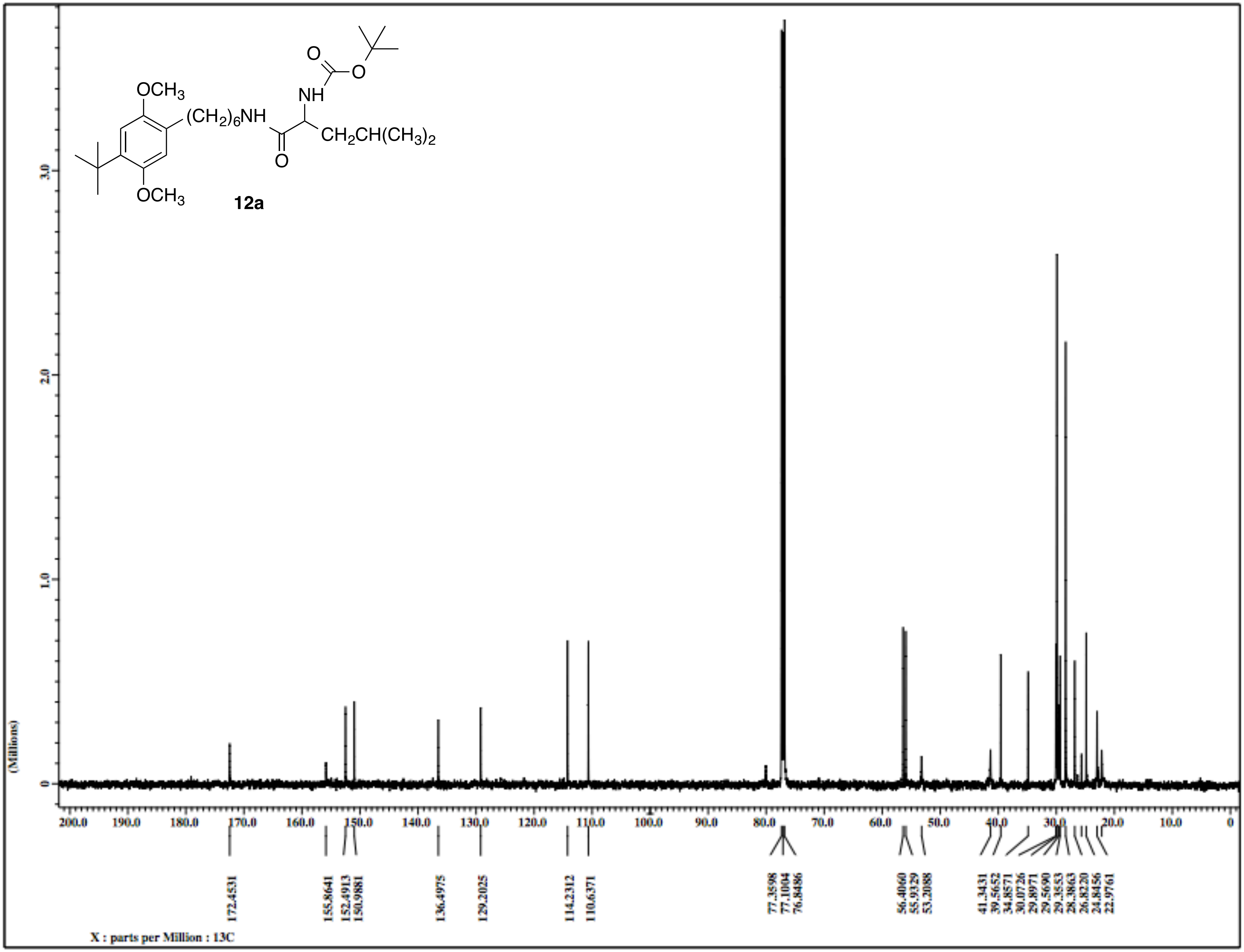
OCH3
(CH2)6NH
NH
O
O
O
CH2CH(CH3)2
OCH3
12a
(Millions)
3.0
2.0
1.0
0
200.0 190.0 180.0 170.0 160.0 150.0 140.0 130.0 120.0 110.0 100.0 90.0 80.0 70.0 60.0 50.0 40.0 30.0 20.0 10.0 0
172.4531
155.8641
152.4913
150.9881
136.4975
129.2025
114.2312
110.6371
77.3598
77.1004
76.8486
56.4060
55.9329
53.2088
41.3431
39.5652
34.8571
30.0726
29.8971
29.5690
29.3553
28.3863
26.8220
24.8456
22.9761
X : parts per Million : 13C

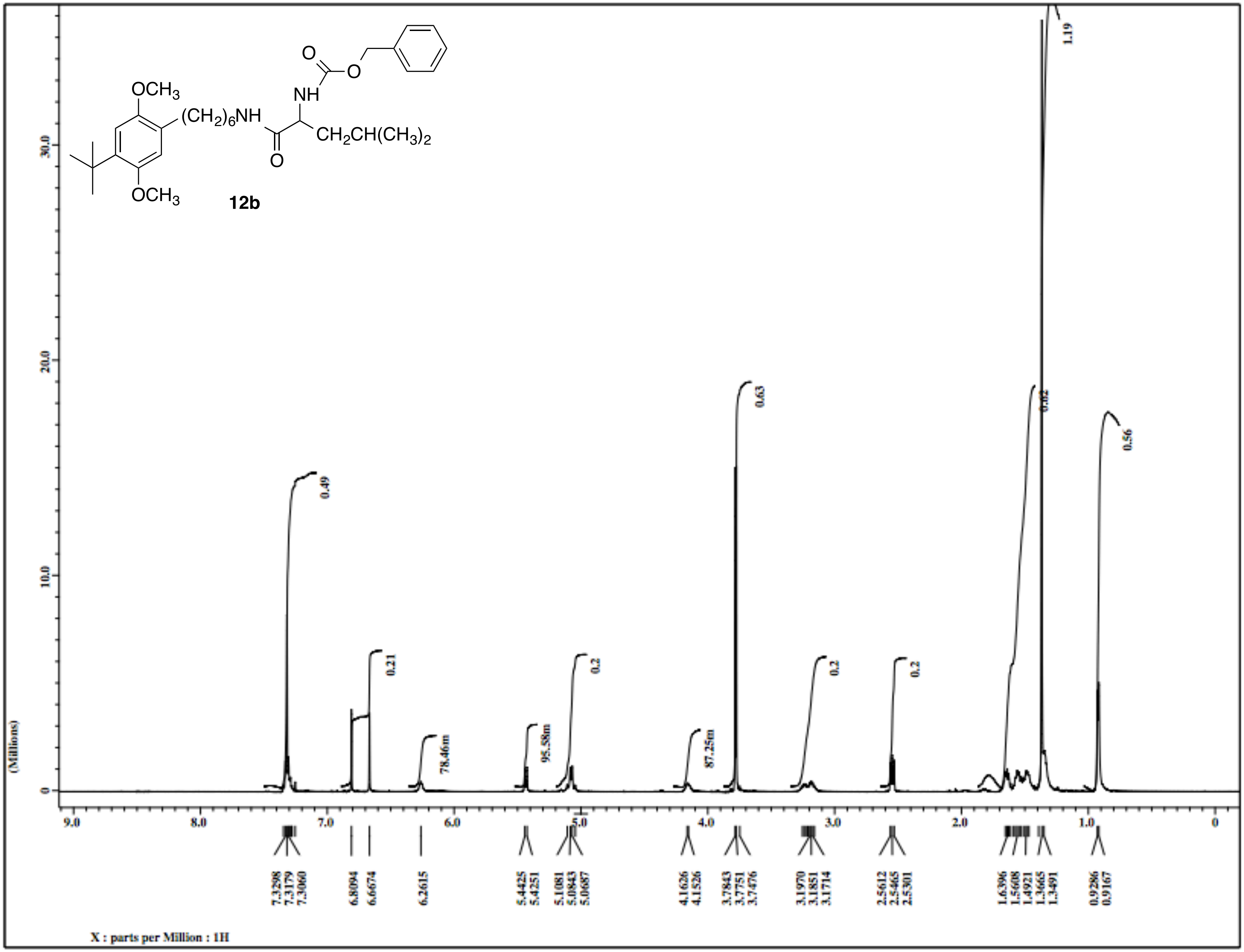

OCH3
(CH2)6NH
NH
O
O
O
CH2CH(CH3)2
OCH3
12b
(Millions)
30.0
20.0
10.0
0
0.49
0.21
78.46m
95.58m
0.2
87.25m
0.63
0.2
0.2
0.82
1.19
0.56
9.0
8.0
7.0
6.0
5.0
4.0
3.0
2.0
1.0
0
7.3298
7.3179
7.3060
6.8094
6.6674
6.2615
5.4425
5.4251
5.1081
5.0843
5.0687
4.1626
4.1526
3.7843
3.7751
3.7476
3.1970
3.1851
3.1714
2.5612
2.5465
2.5301
1.6396
1.5608
1.4921
1.3665
1.3491
0.9286
0.9167
X : parts per Million : 1H

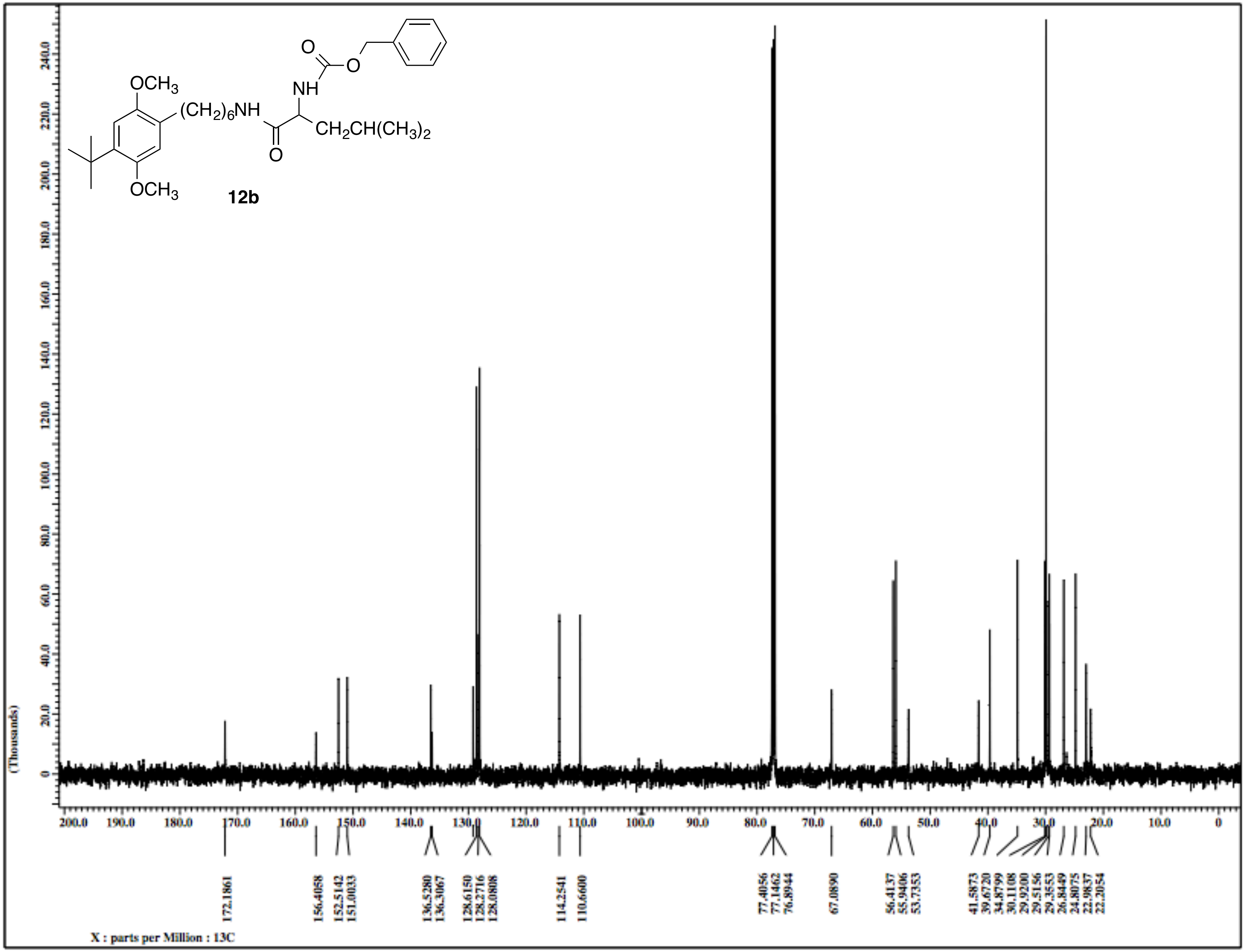

OCH3
(CH2)6NH
NH
O
O
O
CH2CH(CH3)2
OCH3
12b
(Thousands)
172.1861
156.4058
152.5142
151.0033
136.5280
136.3067
128.6150
128.2716
128.0808
114.2541
110.6600
77.4056
77.1462
76.8944
67.0890
56.4137
55.9406
53.7353
41.5873
39.6720
34.8799
30.1108
29.9200
29.5156
29.3553
26.8449
24.8075
22.9837
22.2054
X : parts per Million : 13C

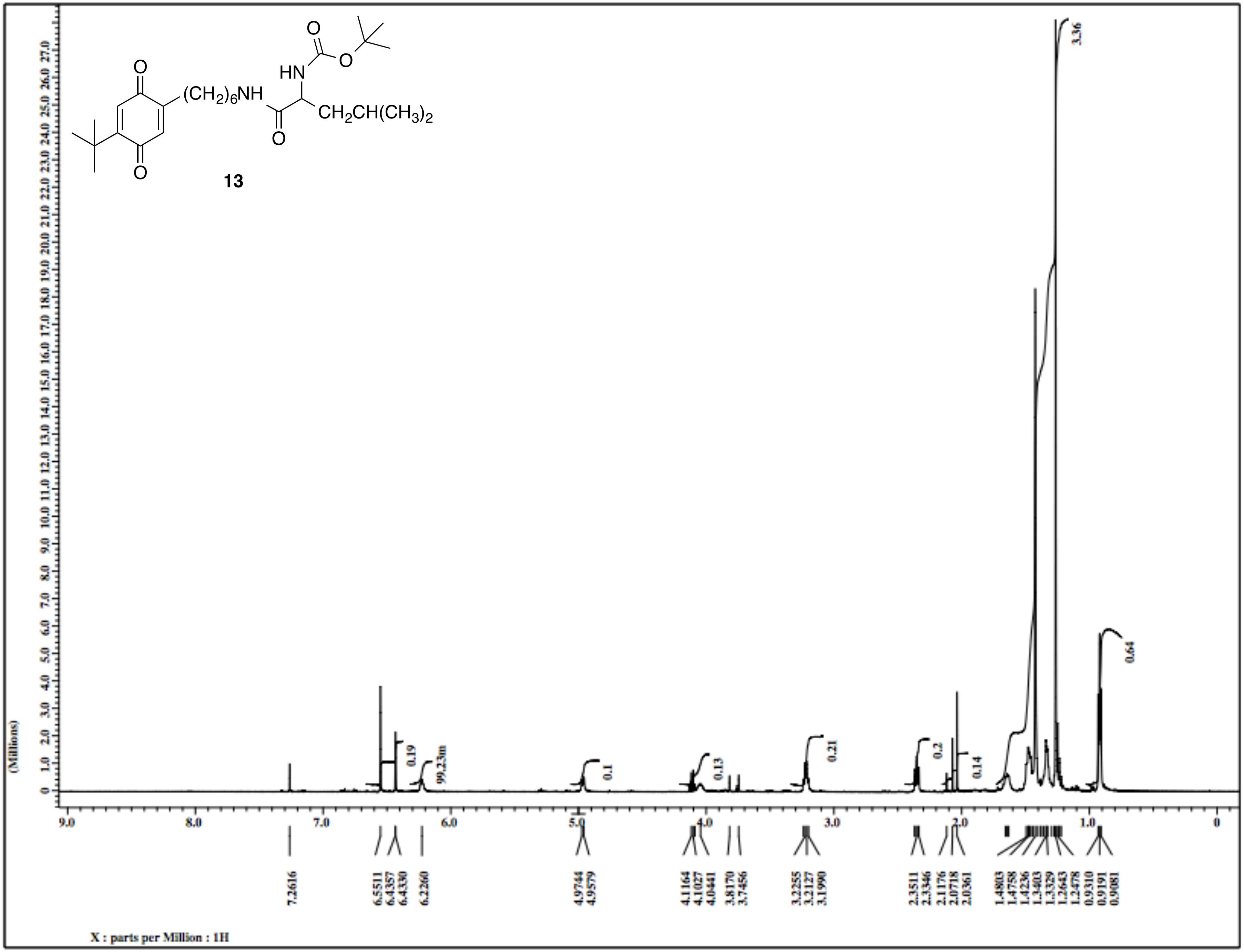
(CH2)6NH
HN
CH2CH(CH3)2
13
3.36
0.64
0.19
99.23m
0.1
0.13
0.21
0.2
0.14
(Millions)
X : parts per Million : 1H
7.2616
6.5511
6.4357
6.4330
6.2260
4.9744
4.9579
4.1164
4.1027
4.0441
3.8170
3.7456
3.2255
3.2127
3.1990
2.3511
2.3346
2.1176
2.0718
2.0361
1.4803
1.4758
1.4236
1.3403
1.3329
1.2643
1.2478
0.9310
0.9191
0.9081

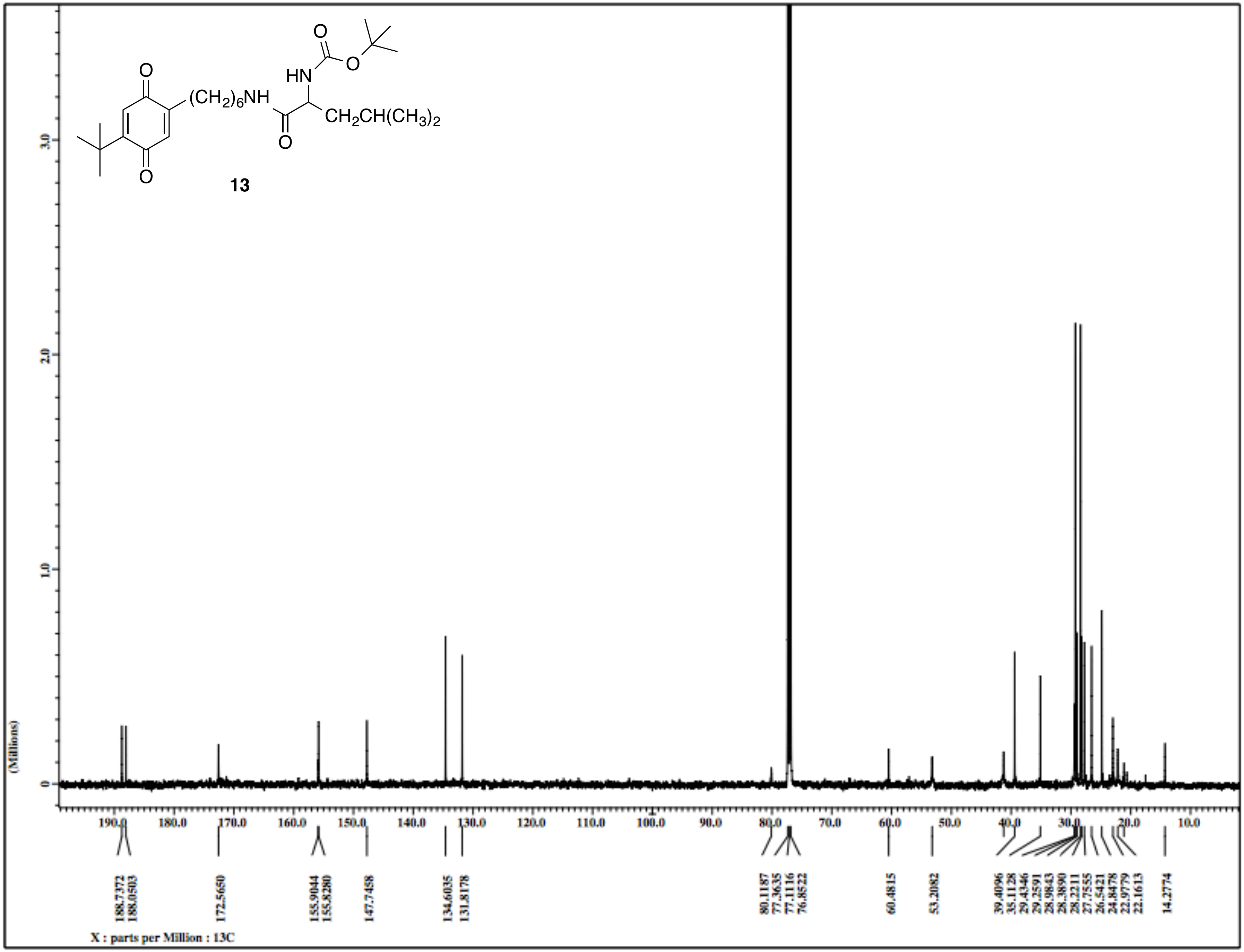
(CH2)6NH
HN
CH2CH(CH3)2
13
(Millions)
X : parts per Million : 13C
188.7372
188.0503
172.5650
155.9044
155.8280
147.7458
134.6035
131.8178
80.1187
77.3635
77.1116
76.8522
60.4815
53.2082
39.4096
35.1128
29.4346
29.2591
28.9843
28.3890
28.2211
27.7555
26.5421
24.8478
22.9779
22.1613
14.2774

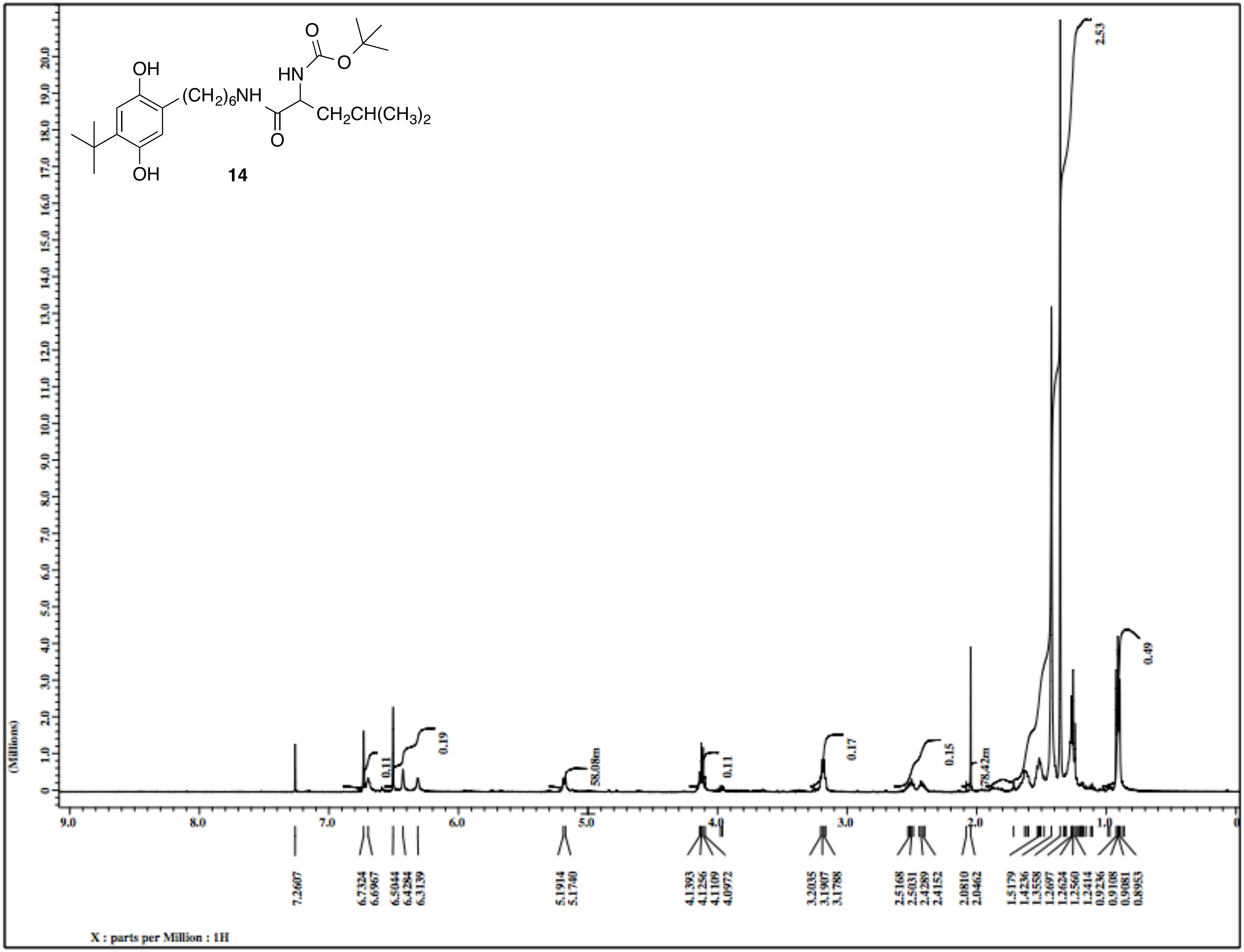

OH
OH
(CH2)6NH
HN
O
O
O
CH2CH(CH3)2
14
(Millions)
X : parts per Million : 1H
7.2607
6.7324
6.6967
6.5044
6.4284
6.3139
5.1914
5.1740
4.1393
4.1256
4.1109
4.0972
3.2035
3.1907
3.1788
2.5168
2.5031
2.4289
2.4152
2.0810
2.0462
1.5179
1.4236
1.3558
1.2697
1.2624
1.2560
1.2414
0.9236
0.9108
0.9081
0.8953
0.11
0.19
58.08m
0.11
0.17
0.15
78.42m
2.53
0.49

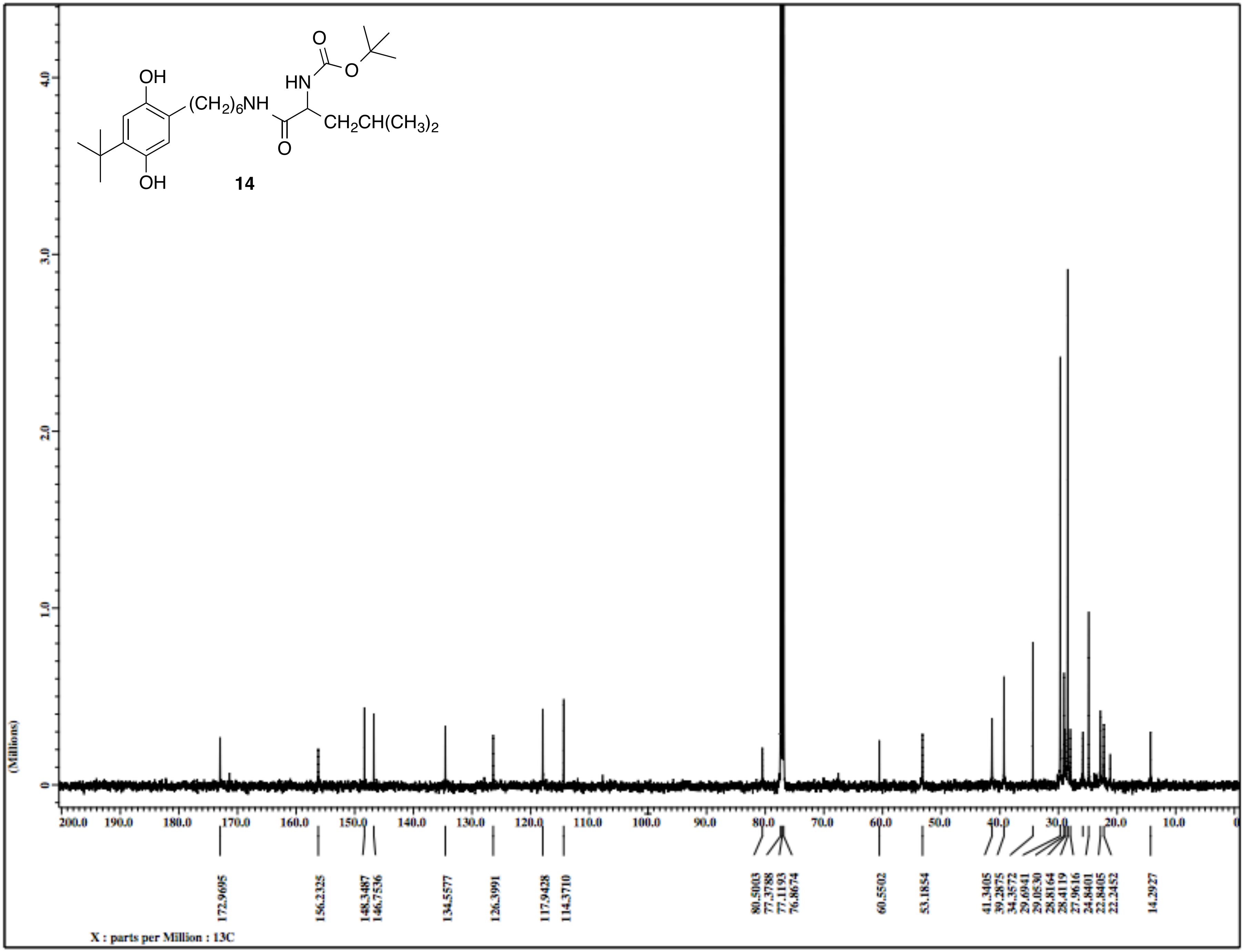

14
(Millions)
X : parts per Million : 13C
172.9695
156.2325
148.3487
146.7536
134.5577
126.3991
117.9428
114.3710
80.5003
77.3788
77.1193
76.8674
60.5502
53.1854
41.3405
39.2875
34.3572
29.6941
29.0530
28.8164
28.4119
27.9616
24.8401
22.8405
22.2452
14.2927
